# Switching of Redox Signaling by Prdx6 Expression Decides Cellular Fate by Hormetic Phenomena Involving Nrf2 and Reactive Oxygen Species

**DOI:** 10.3390/cells11081266

**Published:** 2022-04-08

**Authors:** Bhavana Chhunchha, Eri Kubo, Dhirendra P. Singh

**Affiliations:** 1 Department of Ophthalmology and Visual Sciences, University of Nebraska Medical Center, Omaha, NE 68198, USA; 2 Department of Ophthalmology, Kanazawa Medical University, Kanazawa 9200265, Japan; kuboe@kanazawa-med.ac.jp

**Keywords:** Klf9, Nrf2, Prdx6, oxidative stress, antioxidant

## Abstract

Changes in intracellular reactive oxygen species (ROS) levels due to remodeling of antioxidant defense can affect the status of biological homeostasis in aging/oxidative stress. Peroxiredoxin 6 (Prdx6), an antioxidant gene downstream target for the Nrf2 pathway, plays a role in regulating ROS homeostasis. Using aging human (h) lens epithelial cells (LECs) or *Prdx6*-deficient (*Prdx6^−/−^)* mouse (m) LECs, here we showed that dichlorofluorescein (DCF) oxidation or H_2_O_2_ were strictly controlled by Prdx6. We observed that a moderate degree of oxidative stress augmented Nrf2-mediated Prdx6 expression, while higher doses of H_2_O_2_ (≥100 µM) caused a dramatic loss of Prdx6 expression, resulting in increased DCF oxidation and H_2_O_2_ amplification and cell death. Mechanistically, at increased oxidative stress, Nrf2 upregulated transcriptional factor Klf9, and that Klf9 bound to the promoter and repressed the Prdx6 gene. Similarly, cells overexpressing Klf9 displayed Klf9-dependent Prdx6 suppression and DCF oxidation with H_2_O_2_ amplification, while *Sh*Klf9 reversed the process. Our data revealed that H_2_O_2_ and DCF oxidation levels play a hormetical role, and the Nrf2-Klf9-Prdx6 pathway is pivotal for the phenomena under the conditions of oxidative load/aging. On the whole, the results demonstrate that oxidative hormetical response is essentially based on levels of oxidative triggering and the status of Klf9-Prdx6 pathway activation; thus, Klf9 can be considered as a therapeutic target for hormetic shifting of cellular defense to improve protective resilience to oxidative stress.

## 1. Introduction

In order to survive against oxidative stress produced by internal and external environmental stresses, cells have developed an antioxidant defense system. However, the intensity of oxidative stress can alter antioxidant response-driven survival signaling to the death pathway [1,2,3,4]. Mild or moderate stress can evoke normal physiological functions of cells due to adoptive increased resistance of Nrf2-mediated antioxidant response [1,4], while excessive stress evokes adverse signaling and that may, in turn, lead to age-related disorders, including neurodegenerative disorder, cancer, diabetes, acute respiratory distress syndrome, hypertension, obesity, ocular disorders such as cataractogenesis, glaucoma, etc. [5,6,7,8,9,10]. This biological mechanism is known as the hormetic response (hormesis) to oxidative stress and has been reported in a variety of organisms in responses to drugs or prooxidative electrophilic compounds [11,12,13,14,15,16]. It has been shown that physiological levels of reactive oxygen species (ROS), specifically H_2_O_2_ play a pivotal role in regulating normal biological activities of cells [4,13], and those homeostatic activities depend upon the status of the Nrf2-antioxidants pathway within the cellular microenvironment. Recently, the hormetic effects of ROS and their influences on cell survival/death as well as aging-related diseases have been shown [17,18,19]. These argued that, if not all, some of the Nrf2-regulated antioxidant genes are responsible for the hormetic effects of ROS. Under oxidative stress conditions, cell defense machinery fine-tunes the ROS metabolism along with gene regulation to reset redox homeostasis in favor of cell survival, wherein the role of the Nrf2/ARE (antioxidant response element)-antioxidant pathway is immense. Many antioxidant genes such as phase II enzymes, including Prdx6, are a target for Nrf2/ARE-mediated transcription in response to prooxidants. Superoxide dismutase (SODs), catalase (CAT), glutathione peroxidase (GPxs), thioredoxin reductase (Txnrds), and peroxiredoxins (Prdxs) are primary antioxidant enzymes and play a major role in cytoprotection by regulating ROS homeostasis. Prdx is a relatively new family of antioxidant systems consisting of six members (Prdx1-6), which are classified based on Cysteine residues; typical, 2-Cys, a typical, 2-Cys, and 1-Cys [20]. 1-Cys Prdx6 is a unique member of Prdxs, having GSH peroxidase, calcium-independent intracellular phospholipase A_2_ (aiPLA_2_), and lysophosphatidylcholine acyltransferase (LPCAT) activities. Prdx6 localizes in cytosol, and importantly, this is present in almost all ROS producing organelles, such as lysosomes and lysosomal related organelles, mitochondria, endoplasmic reticulum thereby maintains ROS homeostasis [10,21,22,23]. Nevertheless, Prdx6 is post-translationally modified by a small ubiquitin-like modifier (Sumo) 1, and in response to aging or ROS-driven oxidative stress, its aberrant Sumoylation jeopardizes the integrity of the Prdx6 and protective activity [5]. Interestingly, Sumoylation-deficient Prdx6 (mutated at Sumoylation sites) enhances its protective potential and integrity and protects cells by escaping oxidative stress-induced aberrant Sumoylation signaling [24].

Moreover, the protective response elicited by antioxidants against mild or increased oxidative stress contains a cascade of transcriptional events that entrain the cells accordingly [11,12]. Nrf2 is a key transregulator of genes involved in antioxidation and antioxidant biosynthesis [25,26]. In the inactive form, Nrf2 heteromerizes in the cytoplasm with repressor Keap1 (Kelch-like ECH-associated protein 1). Nrf2 is negatively regulated by Keap1 involving the adapter of a Cul3-ubiquitin ligase complex that directs proteasomal degradation of Nrf2 through ubiquitination [27,28,29,30,31]. Studies suggest that the release of Nrf2 from the Keap1 repressor can be facilitated by Keap1’s posttranslational modification, such as oxidation or covalent modification of some critical cysteine thiols or phosphorylation of critical tyrosine residues [12,32]. However, Nrf2 inducers are chemically different, but they all bear electrophilic characters, and they work by alkylation/oxidation of Cys residues of Keap1 [33]. Recently, it has been shown that oxidative stress regulates Nrf2 stabilization by oxidizing Keap1 with the formation of disulfides [34,35,36,37,38,39]. The ARE is an enhancer site that drives the transcription of phase II detoxification enzymes [40,41]. However, Nrf2 can act as an activator as well as a repressor depending on the magnitude of intracellular ROS induced by stimuli [11,12,13,42]. In this regard, the level of ROS is critical for cell homeostasis. The mechanism involved in the activation of Nrf2 has been found to be related to changes in the redox state and has been proposed to play a part in inducing the hormetic effects depending upon inductors, cell types, and cellular status [43]. Nrf2 can have beneficial as well as harmful effects; Nrf2 activators have been used for cancer prevention and treatment, but on the other hand, studies have shown that constitutive activation of Nrf2 results in cancer cell survival and resistance to anticancer drugs [44,45]. It has been suggested that Nrf2-mediated adaptive responses can be linked to Nrf2’s posttranslational modifications and its interactions with other biological molecules under the conditions of oxidants levels [43,46]. Recently, other investigators are we found that higher concentrations of Nrf2 activators can increase the aberrant nuclear accumulation of Nrf2 with increased expression of transcriptional protein Klf9. In this scenario, we thought that mechanistically, at higher doses, electrophilic stressors can evoke increased levels of ROS generation and that the levels of intracellular ROS might be responsible for Nrf2-mediated aberrant expression of Klf9, which in turn results in Klf9-mediated repression of Prdx6 transcription and further amplification of ROS, and subsequent cell death [11,13]. We postulated that the hormetic effects (a shifting of redox signaling; beneficial to deleterious effect) of ROS depend upon the Nrf2-Klf9-Prdx6 signaling pathway. Indeed, our present study revealed that the Nrf2-Klf9 activation pathway determines cell fate via antioxidant genes, such as Prdx6.

Klf9 (Kruppel-like factor 9) is a ubiquitous transcriptional protein and is also called Basic Transcription Elements Binding (BTEB) protein 1 [47]. This regulates a variety of biological functions, including cell differentiation, cell proliferation, DNA damage, apoptosis, and stress responses [48,49,50], including apoptosis in multiple melanoma cells [51]. Klf9 functions as an activator as well as a repressor depending on the number of GC elements present in the promoter of target genes and cell types [47,48,52]. Studies have shown that gene promoters having multiple or repeated GC-boxes which are activated by Klf9, in contrast to promoters with a single GC-box, are repressed by Klf9 [53]. There are multiple antioxidant genes, such as Prdx6 and TXNRD2, that are found to be repressed by Klf9, resulting in increased ROS-driven cell death [13,54,55]. Importantly, Klf9 is an inducible transcriptional protein and can be stimulated by various stressors. Klf9’s aberrant expression drives stressors-induced oxidative cell death [51]. Klf9-dependent ROS regulation has been reported in melanoma progression in a stage-specific manner [54]. Recently, we have reported that toxic concentrations of SFN dramatically augment cell toxicity via Nrf2-mediated Klf9-dependent suppression of antioxidant gene [11]. This argues that the Nrf2-Klf9 pathway, including suppression of antioxidant genes such as Prdx6, should be involved to regulate this hormesis phenomenon (from survival to death signaling) of oxidative stress. This phenomenon has found further support from recent studies, dictating that aberrant Klf9 upregulation in cells or tissues caused excessive oxidative stress and injuries, while Klf9 knock down protected tissues injuries [56].

Furthermore, the specificity of the cellular stress response can also be regulated by the nature and strength of stimuli and levels of ROS, thereby activating the downstream effectors/signaling [57]. It is worth mentioning that the cellular level of Prdx6 (which is present in all most all ROS producing organelles) can affect cellular physiology by regulating ROS homeostasis. This suggests that the Prdx6 level should be a requisite for the hormetic effects of ROS. Prdx6 is Sumoylated, and Sumoylation of Prdx6 negatively regulates its cellular homeostasis and activity [5]. We surmise that there might be a contribution of Prdx6 Sumoylation, at least in part, in regulating ROS’s hormetic effects. Recently, it has been reported that miR-24 is a regulator of Prdx6, and its downregulation enhances Prdx6 expression with a reduction in ROS accumulation and ROS-mediated damage [58]. However, there is considerable evidence across a wide range of studies that the level of oxidative stress (redox) plays a critical role in biological processes by affecting the transcriptional machinery of antioxidant genes. Nevertheless, there is a gap in the knowledge about the effect(s), and the molecular mechanism(s) involved in the induction of biological responses against different degrees of oxidative stress at the transcriptional level. Furthermore, it is unknown how survival signaling goes awry, and what the specific biomolecule(s) are, specifically transcriptional proteins, are involved during the adverse changes of cell homeostasis, which in turn leads to ROS-driven oxidative cell death and disease state. Moreover, systematic studies at the transcriptional level, specifically pathways examining hormetic effects of oxidative stress, are limited.

Using primary human (h) LECs of variable ages or hLECs exposed to different concentrations of oxidative stress inductor, including Prdx6-deficient mLECs, as a model system(s), here we showed that the hormetic responses to oxidative stress are linked to a degree of activation of Nrf2. We found that excessive oxidative load induced by H_2_O_2_ was directly related to Prdx6 suppression, resulting in a dramatic increase in intracellular ROS and cell death, while lower levels of oxidative stress stimulated adaptive (protective) biological response via Nrf2-Prdx6 pathways. Mechanistically, our data revealed that the aberrant accumulation of Nrf2, paradoxically, resulted in Nrf2 binding to *Klf9* promoter and increasing its expression, which subsequently bound to its repressive Klf9 binding element (RKBE; C^A/G^CCC) of *Prdx6* promoter and suppressed the Prdx6 expression, including ROS amplification and subsequent cell injuries. Together, our results showed that the molecular mechanisms of oxidative stress-mediated hormetical responses that involve the Nrf2-Prdx6 pathway and identified that Klf9’s activation plays a pivotal role. Because aberrant stimulation of Klf9 leads to ROS amplification-dependent cell death, we propose that Klf9 should be a therapeutic target to treat or prevent oxidative stress-associated diseases.

## 2. Materials and Methods

### 2.1. Isolation and Generation of Primary hLECs and SRA-hLECs

Two types of human LECs were used: (1) primary hLECs isolated from different ages of deceased persons and (2) a cell line (SRA01/04) immortalized with SV40. To avoid any confusion, the remaining text will designate the different ages of primary hLECs as primary hLECs and immortalized SRA-hLECs cell lines as hLECs [12].

Primary hLECs were separated and collected from normal eye lenses of deceased subjects or healthy donors of variable ages (16, 33, 54, 66, and 75 y) procured from the Lions Eye Bank, Nebraska Medical Center, Omaha, NE, and National Disease Research Interchange (NDRI), Inc., Philadelphia, PA, USA. In accordance with regulation HHS45CFR 46.102(f), studies involving materials from deceased individuals are not considered human subject research as defined at 45CFR46.102(f) 10(2) and do not need IRB oversight. Briefly, the lens capsule(s) was separated and processed/trimmed before explanting in 35 mm culture dishes coated with collagen IV containing complete DMEM supplemented with 15–20% fetal bovine serum (FBS), with a brief modification [12,21,59,60,61]. The culture explants were trypsinized and re-cultured for hLECs generation. Cell cultures attaining confluency were trypsinized and used for experiments [8,62,63]. Western analysis was carried out using an antibody specific to αA-crystallin to validate the presence of αA-crystallin, a specific marker for LEC identity (data not shown).

SRA-hLECs (hLECs) were derived from 12 infants who underwent surgery for retinopathy of prematurity [64] (a kind gift of late Dr. Venkat N. Reddy, Eye Research Institute, Oakland University, Rochester, MI, USA). Human LECs cell lines (SRA01/04) were immortalized with SV40. These cells were maintained in DMEM with 15–20% FBS, 100 µg/mL streptomycin, and 100 µg/mL penicillin in a 5% CO_2_ environment at 37 °C as described previously [5,65]. To examine the effect of H_2_O_2_, cells were treated with serum-free medium (DMEM medium containing 0.2% BSA) with different concentrations of H_2_O_2_ in DMEM supplemented with 0.2% of BSA. H_2_O_2_ (Catalog no. H1009) and Actinomycin D (Catalog no. A9415) were purchased from Sigma-Aldrich (St. Louis, MO, USA).

### 2.2. Generation and Validation of LECs Isolated from Lenses of Prdx6^+/+^ and Prdx6^−/−^ Mice

All animal experiments followed the recommendations set forth in the “Statement for the Use of Animals in Ophthalmic and Visual Research” by the Association for Research in Vision and Ophthalmology, and studies conducted were approved by The University of Nebraska Medical Center (UNMC). LECs isolated from lenses of Prdx6-targeted mutants (*Prdx6^−/−^*) and wild type (*Prdx6^+/+^*) mice were cultured and maintained in Dulbecco’s modified Eagle medium (DMEM) containing 10% fetal bovine serum (FBS), as described earlier [66]. *Prdx6^−/−^* 129/Sv mice were generated at Harvard Medical School (Boston, MA, USA) under the supervision of Dr. David R. Beier. All animals were maintained under specific pathogen-free conditions in an animal facility.

### 2.3. Real-Time Quantitative Reverse Transcriptase-Polymerase Chain Reaction (RT-qPCR)

The total RNA was extracted from the cultured LECs using the single-step guanidine thiocyanate/phenol/chloroform extraction method (Trizol Reagent, Invitrogen). To examine the levels of Nrf2, Klf9, and Prdx6, 0.5 to 5 micrograms of total RNA were reverse-transcribed to cDNA using Superscript II RNAase H-reverse-transcriptase (Catalog No. 18064071, Invitrogen). qRT- PCR was conducted with SYBR Green Master Mix (Roche Diagnostic Corporation, Indianapolis, IN, USA) in a Roche^®^ LC480 Sequence detector system (Roche Diagnostic Corporation). PCR conditions of 10 min (min) hot start at 95 °C, followed by 45 cycles of 10 s (sec) at 95 °C, 30 s at 60 °C, and 10 s at 72 °C. The primer Sequence was: Human (h) Nuclear factor (erythroid-derived 2)-like 2 (hNrf2), Forward primer: 5′-TGCTTTATAGCGTGCAAACCTCGC-3′; Reverse primer: 5′-ATCCATGTC CCTTGACAGCACAGA-3′; hKlf9, Forward primer: 5′-CTGGTTGCTGGGACTGTAGC-3′; Reverse primer: 5′-GTTTTCCAGCTCCCAAACAG-3′; hPrdx6, Forward primer: 5′-GCATCCGTTTCCACGACT -3′ and Reverse primer: 5′-TGCACACTGGGGTAAAGTCC-3′; hβ-actin, Forward primer: 5′-CCAACCGCGAGAAGATGA-3′ and Reverse primer: 5′-CCAGAGGCGTACAGGGATAG-3′; mouse (m)Prdx6, Forward primer: 5′-TTCAATAGACAGTGTTGAGGATCA-3′ and Reverse primer: 5′-CGTGGGTGTTTCACCATTG-3′; mβ-actin, Forward primer: 5′-CTAAGGCCAACCGTGAAAAG-3′ and Reverse primer: 5′-ACCAGAGGCATACAGGGACA-3′. The relative quantity of the mRNA was obtained using the comparative threshold cycle (CT) method [67]. The expression levels of target genes were normalized to the levels of housekeeping gene β-actin, which remains stable in LECs facing oxidative stress, as an endogenous control in each group [10,11,13,21,68,69].

### 2.4. Protein Expression Analysis

The total cellular extracts of LECs were prepared in ice-cold radioimmune precipitation buffer (RIPA buffer), and Western analysis was carried out as described previously [7,70,71,72]. The membranes were probed with anti-Nrf2 (sc-722, Santa Cruz Biotechnology, Dallas, TX, USA), anti-Nrf2 (ab62352, Abcam^®^, Cambridge, MA, USA), Anti-Klf9 (ab177158, Abcam^®^, Cambridge, MA, USA), Anti-Klf9 (sc-12996, Santa Cruz Biotechnology, Dallas, TX, USA), Anti-Prdx6 antibody (LF-PA0011, Ab Frontier, South Korea), Tubulin (ab44928, Abcam^®^, Cambridge, MA, USA) or β-actin (A2066, Sigma-Aldrich, St. Louis, MO, USA) or Lamin B1 (ab133741, Abcam^®^, Cambridge, MA, USA) as an internal control to monitor levels of protein expressions. After secondary antibody (sc-2354 and sc-2768, Santa Cruz Biotechnology, Dallas, TX, USA) incubation, protein bands were visualized by incubating the membrane with luminol reagent (sc-2048; Santa Cruz Biotechnology, Dallas, TX, USA). Finally, images (bands) were recorded with a FUJIFILM-LAS-4000 luminescent image analyzer (FUJIFILM Medical Systems Inc., Hanover Park, IL, USA).

### 2.5. Quantitation of Intracellular ROS Level by H2-DCF-DA and CellROX^®^ Deep Red Reagent

LECs were cultured in 96-well plates (5000/well) in either the presence or absence of an oxidant. At higher oxidant concentrations, the levels of intracellular ROS were measured after 2–4 h exposure to H_2_O_2_ to establish the connection between levels of ROS and cell death. Note: lethal concentrations will be highly toxic and kill the cells if the cells are incubated for a longer period, and the condition will not provide the correct value of ROS and its connection to cell death, as well as the status of the antioxidant pathway, including the molecular mechanism involved. Thus, in this work, we assessed the biological molecules in cells facing higher concentrations of H_2_O_2_ as reported previously [11,13]. Nonetheless, the time and duration of the LECs treatment with H_2_O_2_ have been indicated in the Figure legends. ROS were quantified using a fluorescent dye, dichlorodihydrofluorescein diacetate (H2-DCF-DA), a nonpolar compound that is converted into a polar derivative (dichlorofluorescein) by cellular esterase after incorporation into cells [10,24,65,72]. On the day of the experiment, the medium was replaced with Hank’s solution containing 10 µM H2-DCF-DA dye. 30 min later, intracellular fluorescence was detected at excitation (Ex) at 485 nm/emission (Em) at 530 nm by a Spectra Max Gemini EM (Mol. Devices, Sunnyvale, CA, USA).

ROS levels were assayed following the company’s protocol (CellROX^®^ Deep Red Oxidative Stress Reagent, Catalog No. C10422) and as described in our published protocol [6]. For the Klf9 overexpression assay, hLECs were transfected with pEGFP-Vector or pGFP-Klf9 plasmid. Then, 48 h later, transfectants were exposed to H_2_O_2_ as indicated in figure legends. For the Klf9 knock down assay, LV *Sh*-control and LV *Sh*-Klf9 infected hLECs (5 × 10^3^) cells were seeded in a 96-well plate. Then, 24 h later, these cells were treated with increasing concentrations of H_2_O_2_. 2–3 h of H_2_O_2_ exposure, CellROX deep red reagent was added with a final concentration of 5 µM and were incubated at 37 °C for 30 min. A medium containing CellROX deep red reagent was removed and fixed with 3.7% formaldehyde. Then, 15 min later, the fluorescence signal was recorded at Ex 640 nm/Em 665 nm with Spectra Max Gemini EM (Mol. Devices, Sunnyvale, CA, USA).

### 2.6. Measurement of H_2_O_2_ Generation in Lens Epithelial Cells

LECs were cultured in 96-well plates. At a predefined time as indicated in figure legends (mostly within 2 h or onwards depending upon oxidant concentrations of oxidant exposure). H_2_O_2_ levels in the cells were measured using an Amplex™ Red Hydrogen Peroxide/Peroxidase Assay Kit (Catalog number A22188, Invitrogen) according to the manufacturer’s protocol and published report [73]. In brief, cells were washed with Krebs-Ringer Solution (Catalog number J67591.K2, Thermo Fisher Scientific, Waltham, MA, USA). Then 100 µL reaction mixture (50 µM Amplex Red reagent and 0.1 U/mL HRP in Krebs-Ringer phosphate) was added. The generation of H_2_O_2_ was measured at different time intervals. For the negative control, 20 µL of Krebs-Ringer phosphate was added to a 96-well containing cells before the addition of a prewarmed 100 µL reaction mixture. To prepare the H_2_O_2_ standard curve, 50 µL of different concentrations of H_2_O_2_ (0–10 µM) prepared in a 1× reaction buffer was added to the 96-well plate, and the reaction was started by adding 50 µL of Amplex Red reagent/HRP working solution (100 µM Amplex Red reagent and 0.2 U/mL HRP in 1X reaction Buffer) and incubated at room temperature in the dark. Reading was recorded at absorbance, ~560 nm.

### 2.7. Cell Survival Assay (MTS Assay)

A colorimetric MTS assay, The CellTiter 96^®^ AQueous One Solution Cell Proliferation Assay (Promega, Madison, WI, USA), was conducted to determine cell viability as described previously [21,65,74]. The assay was carried out by the addition of MTS reagent to 96-well containing cells. The A_490_ nm (O.D.) value was recorded after 2 h with a plate reader, Spectra Max Gemini EM (Mol. Devices, Sunnyvale, CA, USA). Results were normalized with the absorbance of the untreated control(s).

### 2.8. Lentiviral (LV) Infection

copGFP control lentiviral particles (LV *Sh*-Control, sc-108084), BTEB1 (Klf9)/GFP *Sh*RNA (LV *Sh*-Klf9, sc-37716-VS), and Prdx6/GFP ShRNA (LV *Sh*-Prdx6, sc-62896-VS) were purchased from Santa Cruz Biotechnology, Dallas, TX, USA. hLECs were infected with these reagents following the company’s protocols [11]. Briefly, the hLECs were cultured in a 6-well plate in a complete medium. Then, 24 h later, the media was replaced with a medium containing polybrene (sc-134220, Santa Cruz Biotechnology, Dallas, TX, USA). Then these cells were infected by adding the *Sh*-Control, *Sh*-Prdx6, and *Sh*-Klf9 lentiviral particles to the 6-well plate and were incubated overnight. For stable selection, the infectants were treated with puromycin dihydrochloride (sc-108071, Santa Cruz Biotechnology, Dallas, TX, USA). These stable infectants, LV *Sh*-Control, LV *Sh*-Prdx6, or LV *Sh*-Klf9, were used for the experiments.

### 2.9. Eukaryotic Plasmids

pEGFP-C1 plasmid vector for eukaryotic expression was purchased from Clontech (Palo Alto, CA, USA). pGFP-Klf9 (pCMV-AC-GFP-Klf9) plasmid was purchased from OriGene (RG210147, OriGene Technologies, Inc., Rockville, MD, USA). pcDNA3-EGFP-C4-Nrf2 (pEGFP-Nrf2) plasmid were purchased from Addgene (Watertown, MA, USA). Control shRNA (sc-108060) and Nrf2 *Sh*RNA (sc-37030-SH) plasmids were purchased from Santa Cruz Biotechnology. For experimentation, LECs were transfected with Control *Sh*RNA or Nrf2 *Sh*RNA using the Neon Transfection System (Invitrogen, Waltham, MA, USA).

### 2.10. Extraction of Nuclear and Cytosolic Fraction

The nuclear extract was prepared as described earlier [68,72,75]. Briefly, LECs (1 × 10^6^) cultured in 100-mm plates were washed with cold phosphate-buffered saline (pH 7.4). Cells were collected by centrifugation, and then resuspended in 5 pellet volumes of cytoplasmic extraction buffer [(10 mM HEPES (adjusted pH at 7.9), 10 mM KCl, 0.1 mM EDTA, 0.4% (*v/v*) Nonidet P-40, 0.5 mM phenylmethylsulfonyl fluoride (PMSF), 1 mM DTT and Protease inhibitor]. After a brief incubation on ice and centrifugation (4 °C) at 10,000 rpm for 10 min, the cytoplasmic extract was collected. Then, pellet containing the nuclei were resuspended in nuclear extract buffer [(20 mM HEPES (adjusted pH at 7.9), 0.4M NaCl, 1 mM EDTA, 10% (*v/v*) glycerol, 1 mM DTT, 0.5 mM PMSF and Protease Inhibitor] and incubated for 2 h at 4 °C. Finally, the extract was spun down at 14,000 rpm for 15 min, and the extract was collected and stored at −80  °C.

### 2.11. Transcription Factor Nrf2 Activation Assay

Nrf2 activation assay was carried out as described in the company’s protocol (TransAM Nrf2 Transcription Factor Assay Kit, Cat No. 50296, Active motif, Carlsland, CA, USA) and as described in our published protocol [11,67]. Briefly, 10 µg of nuclear extract (up to 10 µL diluted with complete lysis buffer) prepared from H_2_O_2_ exposed LECs was added to the strips well following the addition of 40 µL binding buffer containing 20 pmol of the wild-type or mutated consensus oligonucleotide to each well. The plate was incubated for 1 h at room temperature (RT). Then, 100 µL primary antibody (1:1000 dilution) was added after 3 washes and then incubated at RT for 1 h. 100 µL of diluted anti-rabbit HRP conjugated antibody (1:1000 dilution) was added and incubated for 1 h at RT. Then, 100 µL of developing solution was added to wells after washing and incubated for 2 to 10 min in the dark. Finally, 100 µL of stop solution was added, and Optical Density (O.D.) was measured at 450 nm.

### 2.12. Chromatin Immunoprecipitation (ChIP) Assay for DNA-Protein Binding In Vivo

The ChIP assay was conducted with the ChIP-IT^®^ Express (Cat. No. 53008; Active Motif, Carlsbad, CA, USA) according to the manufacturer’s instruction(s) as well as following our published protocol [11,12,65,67]. Antibodies used were control IgG, an antibody specific to Klf9 (Catalog No. 701888, Thermo Scientific) and Nrf2 (Catalog No. Ab180845, Abcam). RT-PCR amplification was performed with 5 μL of DNA sample. The program applied for amplification was 3 min for 94 °C (denaturation), 20 s at 95 °C, 30 s at 59 °C, and 30 s at 72 °C for 36 cycles in 25 μL reaction volume (RT-PCR). The data obtained with RT-PCR was resolved onto 1.5% agarose gel and the band was visualized under UV, and images were captured. The data obtained from qPCR is presented as a histogram. Primers used are noted below.

#### 2.12.1. Mouse Klf9 Promoter ChIP Primers

Klf9 ARE3:
Forward primer: 5′-CGCTAGAGTTACGAAACAGGG-3′;
Reverse primer: 5′-GAAAGGCCATCCGTTCATGC-3′
Klf9 ARE4:
Forward primer: 5′-GCGCCAGCACCCGGCCGAACC-3′;
Reverse primer: 5′-GCTGGTGTTGCTGTCCCTGG-3′

#### 2.12.2. Human Prdx6 Promoter ChIP Primers

Prdx6 RKBE1:
Forward primer: 5′-GCTGTGCGAAGCCGCCGCA-3′;
Reverse primer: 5′-GAAGCTTGAGGATGCGCCA-3
Prdx6 RKBE2:
Forward primer: 5′-GTAGTCGAGCAGTCACTCCA-3′;
Reverse primer: 5′-GAAGGAAGAGGAACGCGGCAG-3
Prdx6 RKBE3:
Forward primer: 5′-GGTTCATAACAAACAGAAAGG-3′;
Reverse primer: 5′-AGCCCAGCTACGATGAACTG-3
Prdx6 RKBE4:
Forward primer: 5′-GTCTGTCACCGGTTTCCCTT-3′;
Reverse primer: 5′-GAGACCTACTGTGTGCAGGT-3
Prdx6 RKBE5:
Forward primer: 5′-CAGAGCACCTACCGTGAGCT-3′;
Reverse primer: 5′-GAAGGGAAACCGGTGACAGA-3

### 2.13. Preparation of Mouse Klf9 Promoter-Linked to Chloramphenicol Acetyltransferase (CAT) Reporter Plasmid

The 5′-region of mouse Klf9 gene, spanning from −5856 to +71 bp was isolated from mouse genomic DNA using an Advantage^®^ Genomic PCR Kit (Cat. No. 639103 and 639104, Clontech Laboratories, Inc., Mountain View, CA, USA, 94043). A construct of −5856 to +71 bp was engineered by ligating the DNA fragment into CAT reporter plasmid, basic pCAT vector (Promega) by utilizing the *MluI* and *XhoI* sites. The plasmid was amplified and verified by sequencing as previously described [11]. The primers used for isolating the genomic DNA fragment were as follows: Forward primer with *MluI* site: 5′-AAAA*ACGCGT*GGTCATCGTAGGAAAGATGTGG-3′; and Reverse primer containing *XhoI* site: 5′-AAAA*CTCGAG*CCTACGAGACACTTCTTCCC-3′.

### 2.14. Preparation of Human Prdx6gene Promoter-Fused to Chloramphenicol Acetyltransferase (CAT) Reporter Plasmid Vector

The 5′-promoter fragment, spanning from −1559 to +30 bp was isolated from human genomic DNA by using an Advantage^®^ Genomic PCR Kit (Cat. No. 639103 and 639104, Clontech Laboratories, Inc., Mountain View, CA, USA, 94043). The PCR product was cleaned and verified by sequencing as previously described [11]. A fragment containing −1559 to +30 bp with *SacI a*nd *Xho*I was ligated in to basic pCAT vector (Promega) using the *SacI* and *XhoI* sites. Primers containing above restriction sites used were as follows: Forward primer; 5′-GACAGAGTTGAGCTCCACACAG-3′; and Reverse primer; 5′-CACGTCCTCGAGAAGCAGAC-3′.

### 2.15. Site-Directed Mutagenesis (SDM)

SDM was performed with QuikChange^TM^ lightning site-directed mutagenesis kit (Agilent Technologies; Catalog No. 210518), as described in the company’s protocol. Briefly, amino acid exchanges at the Klf9 sites (RKBE 1 and RKBE 3) mutants (RKBE 1; GC to TT and RKBE 3; AC to TT) were produced by point mutations in the human promoter of Prdx6-linked to CAT plasmid as described earlier [11]. To prepare Nrf2/ARE (ARE 3 and ARE 4) sites mutants, base A was changed to C, and base T to G at ARE sequences in the mouse Klf9 promoter-linked to CAT reporter plasmid by using site-directed mutagenesis. The following complementary primers were utilized (changed nucleotides are in red boldface type and underlined). 

#### 2.15.1. Nrf2/ARE SDM Primer

Klf9 promoter, ARE site1 mutant (ARE1-mut; A to C, −1176 to −1164):
Forward primer: 5′-CTCTAAAGCAGAGTCCGGAATCGGGAACC-3′
Reverse primer: 5′-GGTTCCCGATTCCGGACTCTGCTTTAGAG-3′
Klf9 promoter, ARE site2 mutant (ARE2-mut; T to G, −4020 to −4008):
Forward primer: 5′-CAGATGAGGCACTGTTCGGAGAGAGCAAATCTTAC-3′
Reverse primer: 5′-GTAAGATTTGCTCTCTCCGAACAGTGCCTCATCTG-3′
Klf9 promoter, ARE site3 mutant (ARE3-mut; T to G, −5213 to −5203):
Forward primer: 5′-CTGTCCTCAAAGGAACCTGCCTCCTC-3′
Reverse primer: 5′-GAGGAGGCAGGTTCCTTTGAGGACAG-3′
Klf9 promoter, ARE site4 mutant (ARE4-mut; A to C, −5808 to −5798):
Forward primer: 5′-CGATTCCTGCAAAGTCCTCTCCACTCGCAC-3′
Reverse primer: 5′-GTGCGAGTGGAGAGGACTTTGCAGGAATCG-3′

#### 2.15.2. Klf9/RKBE SDM Primer

Prdx6 promoter, RKBE1 mutant (RKBE1-mut; GC to TT, −407 to −403):
Forward primer: 5′-CCCTAAAGCGCGTACTTCCTGCAGAGTCAAACC-3′
Reverse primer: 5′-GGTTTGACTCTGCAGGAAGTACGCGCTTTAGGG-3
Prdx6 promoter, RKBE3 mutant (RKBE3-mut; AC to TT, −700 to −696):
Forward primer: 5′-CTCTGACATAAGGTCTTCCATACTTCTGGGTC-3′
Reverse primer: 5′-GACCCAGAAGTATGGAAGACCTTATGTCAGAG-3

### 2.16. Statistical Analysis

For all data obtained from experiments, statistical analyses were carried out by Student’s *t*-test and by one-way ANOVA. Data were represented as mean ± S.D. of the indicated number of experiments. A significant difference between the control and treatment groups was defined as *p* value of <0.05 and 0.001 for two or more independent experiments.

## 3. Results

### 3.1. ROS/H_2_O_2_-Dependent Biological Response and Cellular Fate Was Linked to Cellular Level of Prdx6

**Figure 1:**
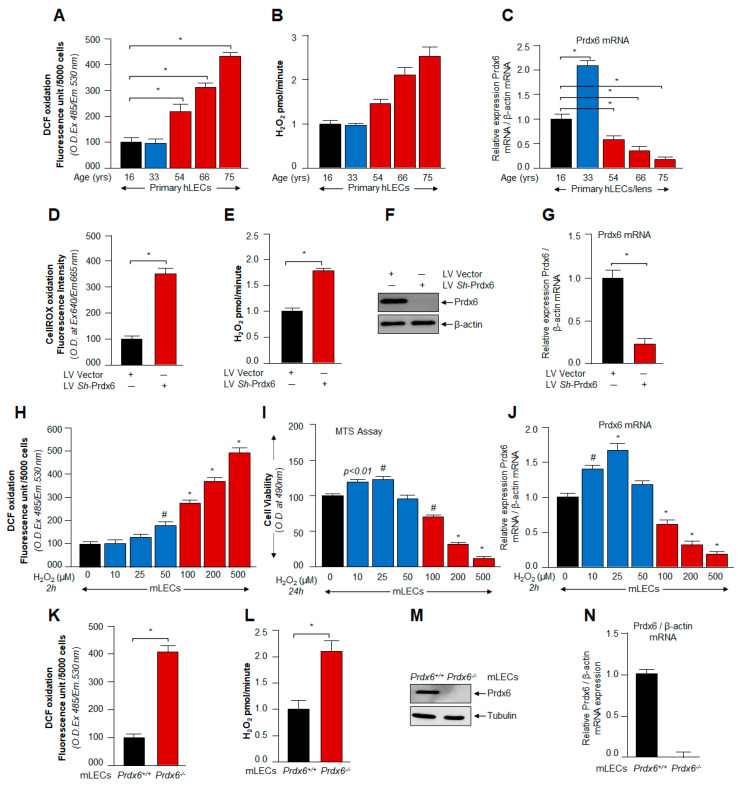
LECs displayed increased ROS levels, H_2_O_2_ generation and reduced level of Prdx6 with advancing age. (**A**–**C**) Increased ROS-induced DCF toxicity and H_2_O_2_ generation with loss of Prdx6 expression was observed in human LECs with aging. (**A**) Intracellular ROS were quantified in primary hLECs of different ages using H2-DCF-DA dye. The data represent the mean ± S.D. from three independent experiments (* *p* < 0.001). (**B**) H_2_O_2_ levels in primary hLECs were measured using Amplex Red reagent and Horseradish Peroxidase (HRP) solution. The data represent the mean ± S.D. from three independent experiments (* *p* < 0.001). (**C**) Total RNA was extracted from LECs isolated from lenses of human individuals of variable age groups and transcribed cDNA was submitted to real time PCR analysis with primers specific to Prdx6. The data represent the mean ± S.D. from three independent experiments (* *p* < 0.001). (**D**–**G**) *Prdx6-*depleted hLECs displayed increased ROS-induced DCF toxicity levels and production of H_2_O_2_ in cells. hLECs were stably infected with GFP (green fluorescence protein) linked lentiviral (LV)-vector or GFP- linked LV *Sh*-Prdx6 as noted in Materials and Methods. (**D**,**E**) Stably infected hLECs with LV-Vector or LV *Sh*-Prdx6 was harvested in 96 well plate. ROS-induced CellROX toxicity (**D**) and H_2_O_2_ generation (**E**) in cells were quantified using CellROX Red reagent dye assay and Amplex Red reagent/Horseradish Peroxidase (HRP) solution, respectively. The data represent the mean ± S.D. from three independent experiments. * *p* < 0.001; LV *Sh*-Prdx6 versus LV-Vector. (**F**,**G**) Cellular extract and RNA were isolated from infected hLECs and examined the Prdx6 protein and mRNA expression using western blot and RT-qPCR analysis with their specific probes, respectively. The data represent the mean ± S.D. from three independent experiments. * *p* < 0.001; LV *Sh*-Prdx6 versus LV-Vector. (**H**–**J**) H_2_O_2_-induced hormetic response on ROS generation, cell viability and antioxidant gene Prdx6 expression in lens epithelial cells. (**H**) mLECs were harvested in 96-well plate and treated with increasing concentrations of H_2_O_2_ as indicated. After 2 h, intracellular ROS were quantified using H2-DCF-DA dye. The data represent the mean ± S.D. from three independent experiments: # *p* < 0.05, * *p* < 0.001. H_2_O_2_ treated versus untreated LECs. (I) MTS assay was carried out to examine cell viability against oxidative stress after 24 h. The data represent the mean ± S.D. from three independent experiments: # *p* < 0.05, * *p* < 0.001. H_2_O_2_ exposed versus unexposed LECs. (**J**) Total RNA was isolated from mLECs exposed to increasing concentrations of H_2_O_2_ as indicated. Prdx6 mRNA expression was analyzed by RT-qPCR using specific primers to Prdx6. The data represent the mean ± S.D. from three independent experiments: # *p* < 0.05, * *p* < 0.001. (**K**–**N**) *Prdx6^−/−^* LECs, a model for aging showed increased levels of ROS accumulation and higher level of H_2_O_2_ generation. (**K**) ROS-induced DCF oxidation levels were quantified in *Prdx6^+/+^* and *Prdx6^−/−^* mLECs using H2-DCF-DA dye. (**L**) *Prdx6*-deficient mLECs showed increased H_2_O_2_ generation as examined using Amplex Red reagent/ Horseradish Peroxidase (HRP) solution. The data represent the mean ± S.D. from three independent experiments (* *p* < 0.001). (**M**,**N**) Total protein and RNA were isolated from *Prdx6^+/+^* and *Prdx6^−/−^* mouse LECs and the protein and mRNA expression were assessed by Western blot (**M**) and real-time PCR (**N**), respectively. Data are the mean ± S.D. of three independent experiments. * *p* < 0.001 vs. control.

In accordance with several reports on the vital influence of ROS, specifically H_2_O_2,_ on cell signaling, from cell survival to cell death, we wanted to understand how the hormetic effect of endogenous ROS are regulated and how the antioxidant pathway plays its role in this context during oxidative stress. Towards this, we first examined levels of Prdx6 mRNA and protein (data not shown) and its correlation with ROS abundance in hLECs-derived from lenses of subjects of different ages. To examine the oxidative status and its effect on primary hLECs and *Prdx6*-deficient mLECs, we monitored the total intracellular ROS (DCF oxidation levels) level of H_2_O_2_ in both LECs. Expression assays and quantitation of intracellular DCF oxidation levels using H2-DCF-DA [5,6] and H_2_O_2_ production using Amplex Red reagent/HRP solution showed an aging-dependent increase in DCF oxidation and H_2_O_2_ levels (Figure 1A,B, 54 y onwards) [5,6,24,72], and that the increasing levels of DCF oxidation and H_2_O_2_ were connected to a significant reduction in the level of Prdx6 mRNA and protein with advancing age (Figure 1A–C). Perhaps unsurprisingly, the data demonstrated that total ROS-mediated DCF oxidation level and the level of H_2_O_2_ had similar patterns of abundance in the LECs. To investigate if there is a direct inverse connection between ROS/cellROx oxidation/H_2_O_2_ levels and Prdx6 expression, we knocked down the Prdx6 gene using shRNA lentiviral specific to Prdx6 in SRA-hLECs (hLECs) and examined the ROS level as well as H_2_O_2_ generation in the cells. We found that, indeed, a significant amplification of CellROX oxidation and H_2_O_2_ levels in *Prdx6*-depleted hLECs as indicated in Figure 1D,E. Figure 1F,G showed successful Prdx6 gene knock down in hLECs. These data pointed to the fact that the biological response of ROS/H_2_O_2_ is tightly regulated through antioxidants, such as Prdx6, and further argued that naturally occurring Prdx6 level is essential to fine-tune ROS levels in favor of cell homeostasis. In addition, results indicated that aging is associated with redox changes due to age-dependent deterioration of antioxidant, Prdx6, thereby leading to ROS accumulation. These observations indicate that hormetical effects of ROS can be controlled by intracellular Prdx6 abundance and provided a base for this study to understand the molecular mechanism(s) involved in the ROS-mediated hormetic response. Here, we would care to mention that we utilized the SRA-hLECs (hLECs), a cell line, to knock down Prdx6 as using primary hLECs in experimentation was cumbersome due to their limited availability.

Furthermore, based upon previously published studies documenting that lower or moderate levels of ROS-induced stress can be beneficial in promoting cellular homeostasis by acting as survival signaling molecules, while excessive oxidative stress further augments ROS-induced oxidative stress with loss of antioxidant response [1,3,11,12,13,15,16,67,76], and coupled with the results of Figure 1 showing suppression of antioxidant protein Prdx6, we intended to examine if there were any connection between cellular abundance of Prdx6 and varying degree of oxidative stress, including modulation in biological responses. To investigate the changes occurring in biological responses to levels of oxidative stress, we induced different levels of oxidative stress in mouse lens epithelial cells (mLECs) by exposing them to different concentrations of H_2_O_2_ (Figure 1H–J). We found that mild/moderate stress resulted in increased Prdx6 expression-dependent regulation of ROS levels in favor of cell health, such as better growth and survival, while increasing doses of H_2_O_2_ (≥100 µM, beyond the threshold levels) led to the failure of ROS homeostasis and cell death with the loss of Prdx6 (Figure 1H–J), suggesting that ROS-mediated hormetic effect was regulated via the cellular abundance of Prdx6. Furthermore, results also revealed an inverse relationship between the ROS and Prdx6 expression (Figure 1H–J). However, we observed increased viability of mLECs exposed to lower concentrations of H_2_O_2_ (Figure 1I). We reasoned that the observed increase in cell viability could be due to increased cell growth, as it is known that H_2_O_2_ can enhance cell proliferation [77]. However, to support the above results, we next utilized *Prdx6*-deficient (*Prdx6^−/−^*) mLECs, a redox-active cell. *Prdx6^−/−^* mLECs are highly susceptible to cell death [10,21]. Perhaps unsurprisingly, we observed a significant increase in ROS levels as well as the presence of higher levels of H_2_O_2_ generation in *Prdx6^−/−^* mLECs (Figure 1K,L), suggesting that Prdx6 plays a pivotal role to regulate ROS homeostasis. Taken together, our data demonstrated that changes in the intracellular ROS during aging or by H_2_O_2_-induced oxidative stress were tightly controlled by an antioxidant defense gene, Prdx6. To unveil the molecular mechanism involved in the hormetic effect of ROS and the contribution of Prdx6 to fine-tune ROS homeostasis, we examined the status of Nrf2 in cells facing different degrees of oxidative stress.

### 3.2. Oxidative Stress-Dependent Nrf2-Klf9 Expression Controlled Prdx6 Abundance-Mediated ROS Homeostasis and Cell Fate

**Figure 2:**
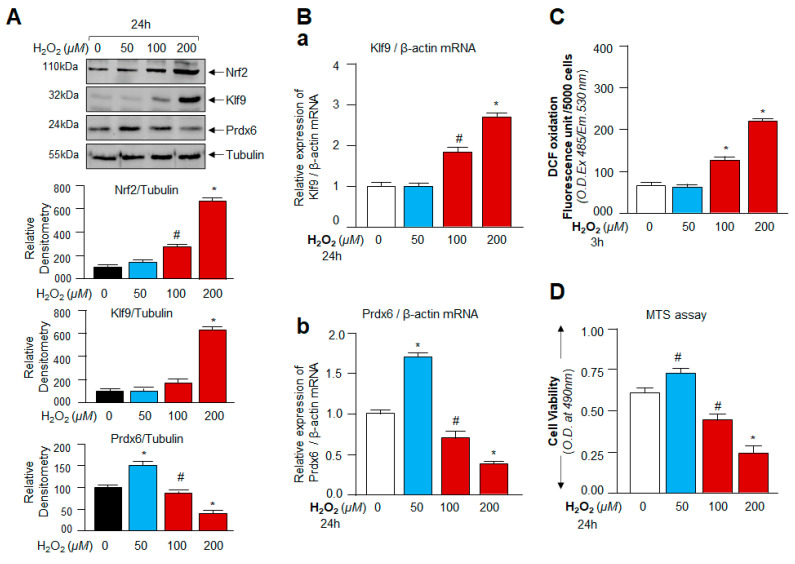
Klf9 and Prdx6 expression were altered in response to oxidative stress and linked to levels of ROS-induced cell death. (**A**,**B**) Mild or moderate oxidative load (50 μM) did not increase Klf9 expression. hLECs were treated with indicated amount of H_2_O_2_ for 24 h, followed by protein (**A**) and mRNA expression (**B**) by Western analysis and real-time PCR quantitation, respectively. Data are the mean ± S.D. of three independent experiments. # *p* < 0.05; * *p* < 0.001 versus control. (**C**) Cultured hLECs were subjected to oxidative stress. Then, 3 h later ROS were measured and presented as DCF oxidation levels. Data are the mean ± S.D. of three independent experiments. * *p* < 0.001 versus control. (**D**) MTS assay was conducted to monitor cell viability against oxidative stress after 24 h. Data are the mean ± S.D. of three independent experiments. # *p* < 0.05; * *p* < 0.001 vs. control.

Knowledge regarding the redox signaling changes and the molecular event(s) in context to ROS-mediated beneficial and/or deleterious signaling(s) (i.e., the hormetic effect of ROS) is extremely limited. However, by reviewing previously published studies [13,51], we found that Klf9 can negatively regulate the antioxidant pathway during excessive stress or cells treated with excessive doses of electrophilic compounds. Thus, we examined expression levels of Nrf2, Klf9, and antioxidant protein Prdx6 and determined their correlation with ROS levels and cell viability in hLECs exposed to mild to high concentrations of H_2_O_2_ as noted in Materials and Methods. Protein blot analysis of total cellular extract prepared from hLECs treated with increasing concentrations of H_2_O_2_ is indicated in Figure 2. The visualization of an immunoblotted membrane with an antibody specific to Nrf2 or Klf9 or Prdx6 revealed a significant increase in Nrf2 levels, and paradoxically the increased expression of Nrf2 was associated with the aberrant expression of Klf9 (not a favorable target for Nrf2 pathway) and a dramatic attenuation of Prdx6 expression in cells treated with ≥100 µM of H_2_O_2_ concentrations. Next, we asked whether the increase in Klf9 protein and reduction in Prdx6 protein occurred at their transcript level. To examine this, we performed RT-qPCR. qPCR analysis (Figure 2B) revealed that hLECs exposed to increasing concentrations of H_2_O_2_ (≥100 µM) displayed increased expression of Klf9 mRNA with a significant decrease in Prdx6 mRNA expression. Conversely, we found that cells exposed to lower concentrations of H_2_O_2_ (<100 µM) had increased Prdx6 expression with only basal expression of Klf9, suggesting that a lower degree of oxidative stress could not induce aberrant Klf9 expression (Figure 2A,B; blue bar).

We next determined the effect of aberrant Klf9 upregulation and its associated depletion of Prdx6 expression on ROS amplification and cellular injuries during different levels of oxidative stress. ROS (Figure 2C) and cell viability (Figure 2D) measurements of hLECs revealed that ≤50 µM of H_2_O_2_ did have distinguishable beneficial effects and provided better cell survival and growth (Figure 2C,D; blue bar). In comparison, cells exposed to ≥100 µM of H_2_O_2_ showed a dramatic augmentation of ROS levels with a subsequent reduction in cell viability (Figure 2C,D; red bars). Together, these observations suggested that physiological redox changes can be governed by the Nrf2-Klf9-Prdx6 pathway and that the pathway could be responsible for ROS-mediated modulation of cell signaling, cell survival, or cell death during oxidant-induced stresses. Because Nrf2 and Klf9 are transcriptional proteins and appeared as the major player during oxidative stress, we wanted to identify how and in what way(s) these factors could play a role in the regulation of Prdx6 and ROS during increased oxidative stress. Thus, to unveil the molecular mechanism(s) involved in the onset of adverse signaling and the roles of Nrf2-Klf9 in the process during excessive oxidative stress, we chose higher doses of the oxidative stress inducer, H_2_O_2,_ to exposed hLECs for our onward experimentation.

### 3.3. Cellular Prdx6 Suppression and ROS Accumulation, and Cell Death Was Connected to Expression and Nuclear Accumulation of Nrf2 and Klf9

**Figure 3:**
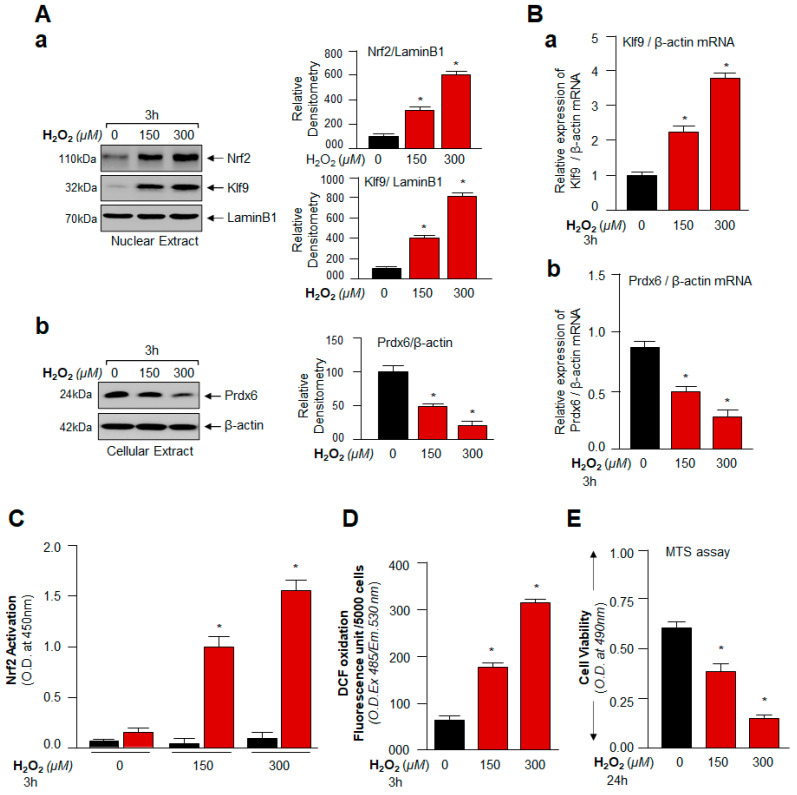
Aberrant Nrf2 and Klf9 expression by excessive oxidative stress was associated with reduced Prdx6 repression, ROS accumulation and increased cell death. (**A**) Cellular and nuclear extract were prepared from hLECs exposed to increasing doses of H_2_O_2_ as indicated in Figure. Samples containing equal amount of protein were immunoblotted using antibody specific to Nrf2 or Klf9 or LaminB1 or Prdx6 or β-actin as shown, and an increased nuclear accumulation of Nrf2 and Klf9 (**Aa**) and reduced in Prdx6 expression (**Ab**) were observed in response to the increased concentrations of H_2_O_2_ treatment. Right adjacent panel reveals densitometry analysis of each protein bands. (**B**) hLECs were exposed to different concentrations of H_2_O_2_. 3 h later total RNA was isolated. Real-time qPCR analysis of Klf9 (**Ba**) and Prdx6 (**Bb**) with their corresponding specific primers. Data are the mean ± S.D. value of three independent experiments. * *p* < 0.001 versus control. (**C**) H_2_O_2_ treated hLECs showed a significant increase in Nrf2 activity. hLECs were treated with different concentrations of H_2_O_2_ for 3 h, and nuclear extract were analyzed for Nrf2-ARE (antioxidant responsive element) binding by ELISA (enzyme-linked immunosorbent assay). Nuclear extracts containing equal amount of protein were processed and assayed for Nrf2 activity with a commercially available kit (Active motif) as described in Materials and Methods. The data represent the mean ± S.D. from three independent experiments. *p* values were determined; H_2_O_2_ treated versus untreated control. * *p* < 0.001. Note: to avoid effect of cell death against excessive oxidative stress, cell extracts were prepared at 3 h of oxidant exposure for the study. (**D**) Cultured hLECs treated with increasing concentrations of H_2_O_2_, 3 h later ROS were measured by H2-DCF-DH dye and data were presented as DCF oxidation levels. Data are the mean ± S.D. of three independent experiments. * *p* < 0.001 vs. control. (**E**) MTS assay was conducted to examine cell viability against oxidative stress after 24 h, and was well corelated to ROS levels. Data are the mean ± S.D. of three independent experiments. * *p* < 0.001 vs. control.

From the above experiments, it was apparent that excessively increased expression of Nrf2 and Klf9 is related to decreased expression of Prdx6 with increased ROS levels and cell death, but it was not clear whether these transcriptional proteins were present in the nucleus to exert their activity. To determine intracellular localization(s) of nuclear Nrf2 and Klf9 and cytoplasmic Prdx6 protein in hLECs in response to higher doses of H_2_O_2_ exposure, we analyzed the nuclear and cytosolic fractions of H_2_O_2_-treated hLECs for 3 h (acute stress) using expression assay (Figure 3A). We observed that Nrf2 and Klf9 migrated at 110 kDa and 32 kDa when immunoblotted with antibodies specific to Nrf2 and Klf9, respectively. Nrf2 and Klf9 in the nuclear fraction of hLECs were dramatically accumulated with increasing concentrations of H_2_O_2_; however, the highest accumulation could be detected with 300 µM of H_2_O_2_. We observed that the increased nuclear accumulation of Nrf2 and Klf9 was directly related to a significant depletion of cytosolic Prdx6 expression (Figure 2 and Figure 3), pointing to the possibility that Prdx6 suppression was Klf9-dependent in hLECs facing excessive oxidative pressure. To determine the fate and status of Klf9, we treated cells with higher doses of H_2_O_2_ (150 and 300 µM) and examined the Klf9 and Prdx6 mRNA expression by RT-qPCR (Figure 3B). However, unexpectedly, we noticed a dramatic inverse correlation between increased Klf9 expression and Nrf2’s target gene Prdx6 mRNA in hLECs facing higher doses of H_2_O_2_. To determine if this inverse correlation was due to dysregulation of Nrf2 activity or due to increased abundance of Klf9, we assessed the Nrf2’s transactivation capacity by using TransAM Nrf2 transcription factor assay (Active motif) using equal amounts of a nuclear extract isolated from H_2_O_2_ exposed or unexposed hLECs. Figure 3C showed a progressive increase in Nrf2-DNA binding activity in response to increasing concentrations of H_2_O_2_, demonstrating that Nrf2 activity is not dysregulated. This finding indicated that there might be involvement of other factor(s), such as Klf9 repressing Prdx6 mRNA as Nrf2 is in an activated form (Figure 3C). We wanted to confirm whether levels of ROS and cell death were increased, as observed in Figure 2, or some other molecular event(s) occurred at extreme oxidative stress (300 µM of H_2_O_2_). However, results revealed that excessive concentrations of H_2_O_2_-induced oxidative stress caused a dramatic increase in intracellular ROS when observed at 3 h of H_2_O_2_ exposure (Figure 3D), leading to increased ROS-dependent increased cell death (Figure 3E). Notably, this injurious process was well correlated with aberrant upregulation of Nrf2 and Klf9 regulation. These results (Figure 2 and Figure 3) served as a base to further explore the role of Nrf2 and Klf9 at the molecular level in the regulation of ROS-mediated hormetical effects. However, we acknowledge that we have used unphysiological concentrations of H_2_O_2_ to treat cells in vitro to explore the plausible mechanism that may occur in the regulation of ROS hormetic responses in vivo since cells cultured in vitro develop adoptive as well as reductive responses and become relatively insensitive and therefore, they require higher concentration(s) of oxidants or other biomolecules to respond [9,10,11,13,72]. However, the in vitro work conducted in the present study should uncover the hormetic effects of ROS. However, since Prdx6 was repressed in response to aberrant Klf9 expression during excessive oxidative stress, next we examined the role of Klf9 on Prdx6 regulation-dependent ROS levels in hLECs over-or under-expressing Klf9.

### 3.4. Overexpression of Klf9 Resulted in Increased ROS Production and Reduced Cells Viability with a Significant Reduction in Prdx6 Expression in hLECs during Oxidative Stress

**Figure 4:**
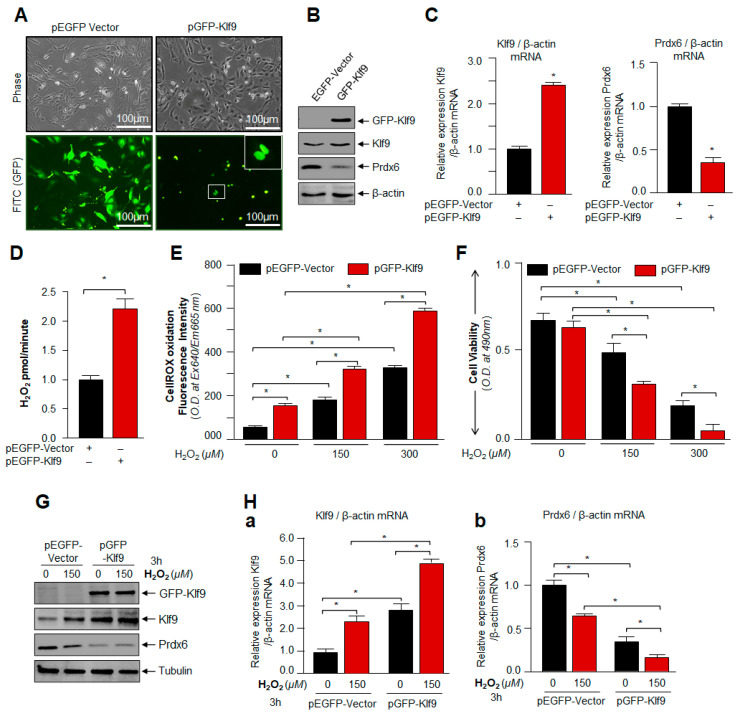
Overexpression of Klf9 generated high production of ROS and H_2_O_2_ with reduced hLECs viability compared to empty vector during oxidative stress. hLECs were overexpressed with pEGFP-Vector or pGFP-Klf9 plasmid. (**A**–**H**) hLECs were transfected with Klf9 plasmid using Neon transfection system and harvested and photomicrograph was taken (**A**). Then, 48 h later, total protein and RNA were isolated from 60mm plates and proceed for Western blot (**B**) and mRNA (**C**) analyses. (**D**) Transfectants were harvested in 96-well plate, and H_2_O_2_ generation of cells were quantified. Data represent the mean ± S.D. of three independent experiments. * *p* < 0.001; pGFP-Klf9 vs. pEGFP-Vector transfected hLECs. (**E**,**F**) To assess effect of H_2_O_2_, transfectants (96-well plate) were exposed to 0, 150 and 300 μM of H_2_O_2_. 3 h of H_2_O_2_ exposure ROS level (E) and at 24 h cell viability (F) was examined. * *p* < 0.001; pGFP-Klf9 vs. pEGFP-Vector transfected hLECs and H_2_O_2_ (150 µM and 300 µM) treated vs. untreated (0 µM) hLECs. (**G**,**H**) LECs overexpressing pGFP-Klf9 were highly sensitive to oxidative stress and showed reduction in Prdx6 expression. hLECs were overexpressed with pEGFP-Vector or pGFP-Klf9 plasmid. 48 h later, complete media were replaced with 0.2% BSA, and cells were exposed to different concentration (0 and 150 μM) H_2_O_2_. After 3 h of H_2_O_2_ exposure, total protein (**G**) and RNA (**H**) were isolated and expression levels were examined by western blot and qPCR, respectively. Data represent the mean ± S.D. of three independent experiments. * *p* < 0.001; H_2_O_2_ treated versus control and pGFP-Klf9 vs. pEGFP-Vector transfected hLECs.

To investigate whether an increase in cellular abundance of Klf9 is, indeed, a cause for Prdx6 suppression as observed during excessive oxidative stress, we extrinsically expressed hLECs with Klf9 plasmid (Figure 4); either with pGFP-Klf9 or pGFP-empty vector plasmid. An equal amount of plasmid DNA was transfected to avoid any effect of DNA concentration on cells. Photomicrographs showed the transfection efficiency (Figure 4A) and no detectable changes in phenotype(s) between the overexpressing Klf9 and/or EGFP-vector-transfected hLECs. A careful analysis of results obtained from western blot (Figure 4B) and qPCR (Figure 4C) of protein extract and total RNA isolated from the transfectants revealed that cells overexpressing Klf9 bore reduced levels of Prdx6 protein and mRNA. To determine the correlation between endogenously generated H_2_O_2_ in cells and the overexpression of Klf9, first, we examined the H_2_O_2_ levels in Klf9 overexpressed hLECs. Quantification of H_2_O_2_ generation in cells by Amplex Red reagent/HRP assay showed significantly elevated levels of H_2_O_2_ production (Figure 4D) in Klf9 overexpressed cells, indicating that Klf9 overexpression enhanced the ROS. Next, we determined the total levels of ROS and cell death of transfectants of the same batch in response to oxidative stress, as indicated in Figure 4A–C. Quantification of ROS (Figure 4E, red bars vs. black bars) by CellROX Deep Red dye and cell viability (Figure 4F, red bars vs. black bars) by MTS assay revealed that Klf9 overexpression caused a significant increase in ROS-induced DCF oxidation levels and cell death, demonstrating Klf9’s involvement in the regulation of ROS via antioxidants, such as Prdx6 in response to an oxidative stressor. Next, we asked the question of if cells expressing Klf9 beyond the threshold levels are responsive and further showed the stimulation of endogenous Klf9 or expression of Klf9 could be inhibited by other cellular pathways in favor of cell survival in response to oxidative stress. To examine this, hLECs overexpressing Klf9 were treated with 150 µM of H_2_O_2_ as indicated in Figure 4G,H. Western analysis and qPCR of total protein and RNA isolated from the transfectants showed further increase in Klf9 and a significant decrease in Prdx6 expression, suggesting there was no dominant active survival pathway, and Klf9 -mediated adverse pathway was dominant. The data demonstrated that the induction of Klf9 and/or its extrinsic overexpression resulted in Klf9-dependent ROS amplification and cell death, which was directly related to the reduced expression of antioxidant genes such as Prdx6. Since other antioxidants failed to protect cells, we argue that here the role of the Nrf2-Klf9-Prdx6 axis is a major determinant of the hormetical effect of ROS and cell fate.

### 3.5. Klf9 Depletion Caused Reduction in ROS Level and Abated Cell Death by Increasing Prdx6 Expression

**Figure 5:**
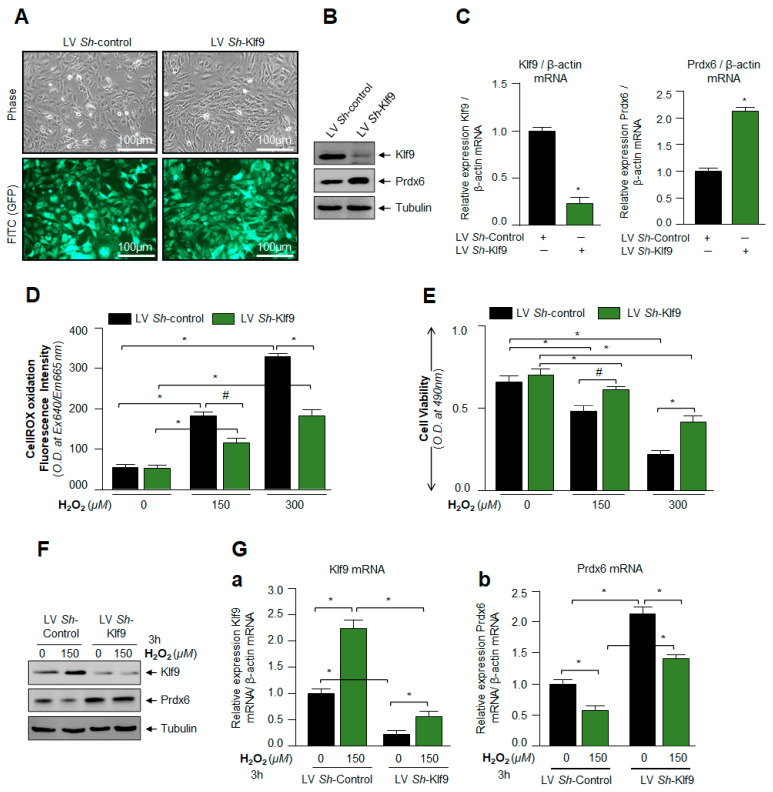
Klf9 gene knock down in hLECs showed elevated Prdx6 expression with reduced ROS and increased hLECs viability against oxidative stress. (**A**–**C**) hLECs were stably infected either with GFP (green fluorescence protein) linked lentiviral (LV) *Sh*-control or GFP linked LV *Sh*-Klf9 as described in Section 2. (**A**) Photomicrograph showing stably lentiviral infected hLECs, left panel: LV *Sh*-Control; right panel: LV *Sh*-Klf9. (**B**) Total cell lysate prepared from lentiviral infected hLECs having equal amount of protein was immunoblotted with antibodies specific to Klf9, Prdx6, and tubulin (loading control). (**C**) Total RNA was isolated from hLECs infected with LV *Sh*-Control or LV *Sh*-Klf9 and RT-qPCR analysis was conducted with Klf9 and Prdx6 specific primers. Data are the mean ± S.D. value of three independent experiments. * *p* < 0.001 vs. control. (**D**,**E**) *Klf9*-depleted hLECs showed reduced ROS levels and increased cell viability. hLECs were stably infected with LV *Sh*-Control or LV *Sh*-Klf9 lentiviral particle following selection procedure. Cells were harvested in 96 well plate. After 24 h, those cells were exposed to 0, 150, and 300 μM of H_2_O_2_. 3 h of H_2_O_2_ exposure ROS intensity (**D**) and at 24 h cell viability (**E**) was examined. # *p* < 0.05, * *p* < 0.001; LV *Sh*-Klf9 vs. LV *Sh*-Control and H_2_O_2_ (150 µM and 300 µM) treated vs. untreated (0 µM) cells. (**F**,**G**) Oxidative stress could not suppress Prdx6 level in *Klf9*-knock down hLECs. hLECs were infected with LV *Sh*-Control and/or LV *Sh*-Klf9 and exposed to H_2_O_2_ as indicated for 3 h. Total protein and RNA were isolated from H_2_O_2_ exposed *Klf9*-depleted hLECs and proceed for western (**F**) and RT-qPCR (**Ga**,**Gb**) for protein and mRNA analyses, respectively. Data are the mean ± S.D. of three independent experiments. * *p* < 0.001; LV *Sh*-Klf9 vs. LV *Sh*-Control and H_2_O_2_ treated vs. untreated hLECs.

The set of outcomes of previous experiments in this study demonstrated that overexpression of Klf9 or induction and cellular accumulation of Klf9 in response to increased ROS-driven oxidative stress was injurious to cells, and it was due to Klf9-dependent repression of antioxidant genes, such as Prdx6. Next, we were interested to know whether Klf9 knock down caused the reduction in ROS levels with increased Prdx6 expression and hLECs viability from oxidative stress as reported for other cell types [13]. To examine this, we depleted Klf9 by stably infecting hLECs using lentiviral (LV) *Sh*-Control or LV *Sh*-Klf9. *Klf9*-depleted hLECs did not show any detectable morphological changes and were indistinguishable from LV *Sh*-Control (Figure 5A). Protein and mRNA data using immunoblot and qPCR demonstrated a remarkable increase in Prdx6 protein (Figure 5B) and mRNA (Figure 5C) expression, indicating that Klf9 was directly involved in the regulation of Prdx6. Next, we examined if *Klf9*-depleted hLECs could confer resistance against an increased concentration of H_2_O_2_, as shown in Figure 5D,E. Quantitation of ROS by CellROX Deep Red reagent and cell viability by using MTS assay demonstrated that, indeed, *Klf9*-deficient hLECs conferred resistance against oxidative stress. Furthermore, we also intended to know the status of Klf9 and Prdx6 in *Klf9*-depleted cells in response to oxidative stress. We did this experimentation to confirm whether Klf9 inhibitors, such as ShRNA-specific to Klf9, could be a useful strategy to block its aberrant expression-mediated ROS amplification and to restore antioxidant genes suppression, such as Prdx6. We observed that *Klf9*-depleted cells had an increased ability to survive against H_2_O_2,_ as shown in Figure 5D,E, and that this was linked to levels of Prdx6 protein (Figure 5F) and mRNA (Figure 5G). Thus, we anticipate that the Nrf2-Klf9 axis plays a central role in controlling ROS-mediated hormetic response by altering antioxidant genes expression, such as Prdx6.

### 3.6. Klf9 Gene Bore Nrf2 Binding Sequences, ARE, and Nrf2/ARE Binding Increased with Increase in Oxidative Stress

**Figure 6:**
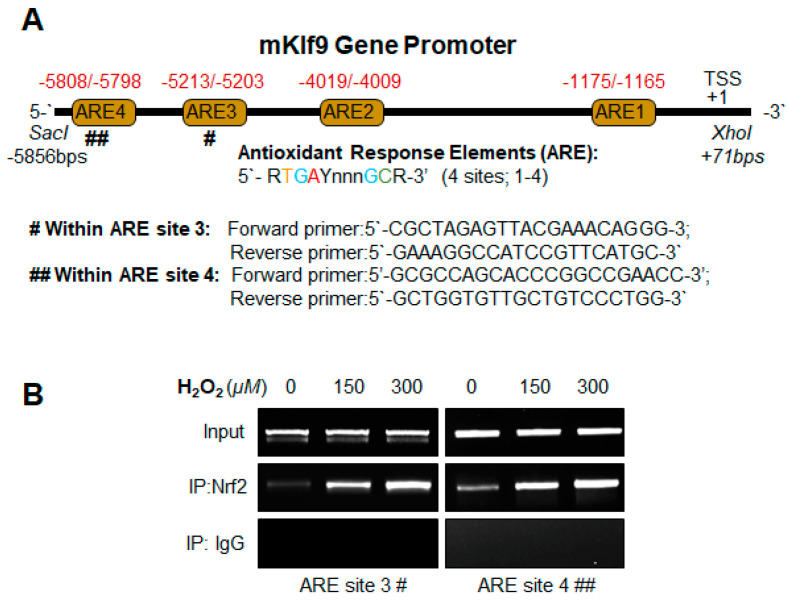
Nrf2 binding was increased to ARE sites present in *Klf9* promoter in response to excessive oxidative stress. (**A**) Top panel displaying the presence of four ARE sites in *Klf9* promoter and ChIP-qPCR primers consisting of ARE3 and ARE4. (**B**) In vivo DNA binding assay disclosed Nrf2/ARE interaction. hLECs treated with different concentrations of H_2_O_2_ (150 and 300 µM) for 3 h. ChIP assay was carried out with Nrf2 and control IgG antibodies. Pulled DNA fragments were subjected to RT-qPCR analysis. DNA fragments present in the immunoprecipitation were amplified with primers that specifically recognized a fragment of *Klf9* promoter containing ARE site three and ARE site four as indicated. 10% chromatin was used as input. As a negative control, ChIP with control IgG was used. # and ## denotes primer pairs used for ChIP-RT-PCR.

Based on the results obtained from the experiments showing changes at the transcript level (Figure 1, Figure 2, Figure 3, Figure 4 and Figure 5) in response to oxidative stress, we posited the involvement of transcriptional machinery and conducted experiments to unveil the mechanism of Nrf2 and Klf9-dependent regulation of antioxidant gene, Prdx6, and ROS levels during oxidative stress. Recent studies have shown that the *Klf9* promoter bears four putative Nrf2/ARE (antioxidant response elements) binding sites [11,13]. However, sites one and two of Nrf2/ARE were found to be unresponsive (data not shown), while sites three and four of Nrf2/ARE were responsive. It is worth noting that Nrf2 is not a bona fide regulator of Klf9; paradoxically, Klf9 can be transcriptionally regulated by Nrf2 in response to higher doses of electrophilic compounds [11,13]. Thus, in this study, we wanted to determine whether Klf9 could be a target gene of Nrf2 in response to higher oxidative stress. To this end, we first examined if oxidative-stress induced Nrf2 binding to ARE present in the *Klf9* promoter as observed in previous studies. To this end, hLECs were treated with different concentrations of H_2_O_2_, and a ChIP-RT-PCR assay was performed to examine the occupancy of Nrf2 on ARE sites present in the Klf9 gene promoter by using antibodies specific to Nrf2 and control IgG as described earlier [11,65,67]. The immunoprecipitated chromatin products were processed and subjected to RT-PCR with specific primers designed for Nrf2/ARE binding sites three and four in *Klf9* promoter regions, as indicated in Figure 6A. As shown in Figure 6B, we observed an increased enrichment of Nrf2 on both sites (AREs 3 and 4), and the enrichment of Nrf2 at ARE sequences was dependent on concentrations of H_2_O_2_. The data analyses demonstrated that higher doses of H_2_O_2_ aberrantly and unphysiologically regulated the Nrf2 expression and its accumulation into the nucleus and that in turn led to Nrf2/ARE interaction of the *Klf9* gene (Figure 6B). There was no binding with IgG control, demonstrating the specificity of Nrf2 binding at ARE sequences. Taken together, the data demonstrated that the upregulation of *Klf9* might be linked to the direct binding of Nrf2 to ARE present in the Klf9 gene promoter during increased oxidative stress.

### 3.7. Nrf2 Upregulated Klf9 Transcription through ARE Binding and the Transcriptional Activity Was Oxidative Load-Dependent

**Figure 7:**
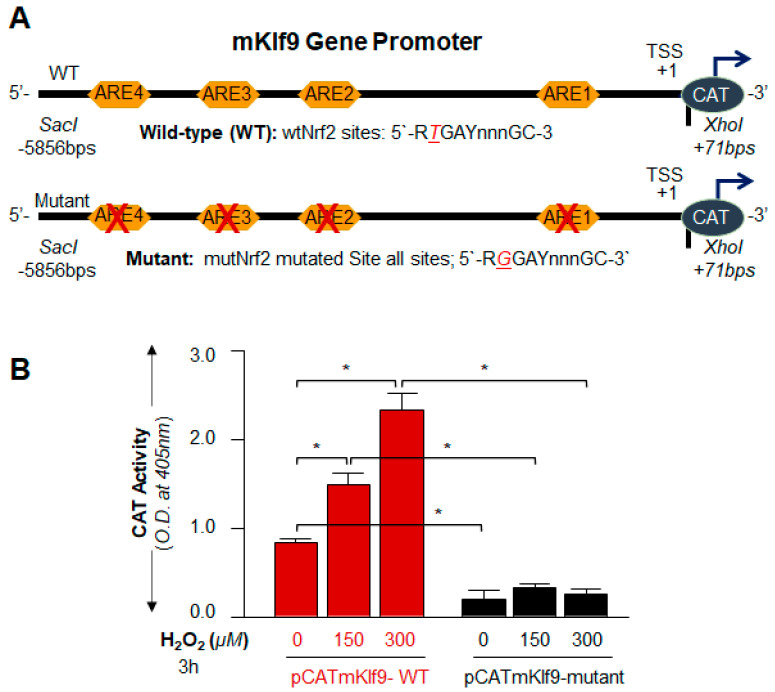
Oxidative load-dependent increase in Klf9’s transcriptional activity through ARE. (**A**) Top panel: diagrammatic illustration of *Klf9* promoter containing predicted ARE sites with wild type (WT) ARE and mutant sequence (mutated using SDM). (**B**) hLECs were transfected with WT mouse *Klf9* promoter (−5856/+71) or its mutant at ARE sites 1 to 4. 48 h later transfectants were treated different concentrations of H_2_O_2_ for 3 h as shown, and *Klf9* Promoter activity was evaluated. Data are the mean ± S.D. of three independent experiments. Klf9-mutant versus Klf9 WT plasmid linked to CAT vector transfected hLECs and H_2_O_2_ treated versus untreated hLECs; * *p* < 0.001.

Next, we looked at if the observed physical binding of Nrf2 to its responsive elements, ARE in the regulatory regions of the *Klf9* promoter was functional and could activate Klf9 transcription. Toward this, Figure 6 results of in vivo DNA binding experiment led the way to design the CAT reporter plasmids of *Klf9* promoter: (i) pCAT-Klf9 wild-type (WT) and (ii) pCAT-Klf9 mutant(s) at all sites (mutant). To determine H_2_O_2_-induced changes in Klf9 transcription, we transfected hLECs with pCAT-Klf9-WT or its mutant plasmid, along with EGFP-empty vector plasmid, and then these transfectants were treated with an increasing concentration of H_2_O_2_ to induce oxidative stress as indicated in Figure 7. We observed that the *Klf9* promoter activity was drastically reduced in the mutant Klf9 promoter where ARE sites were disrupted (Figure 7B; black bars) compared to the WT Klf9 promoter. These results documented that Klf9 is transcriptionally regulated by a Nrf2/ARE mechanism, and the increase in the transcriptional activity was dependent on oxidative levels. These results indicated that the levels of ROS could be controlled via Klf9 regulation of the antioxidant gene, Prdx6, and we postulated that Prdx6 could be a target gene for Klf9.

### 3.8. In Vivo DNA Binding Assay Disclosed That Oxidative Stress Level Stimulated Klf9 Binding to Its New Target Gene, Prdx6 in Dose-Dependent Fashion

**Figure 8:**
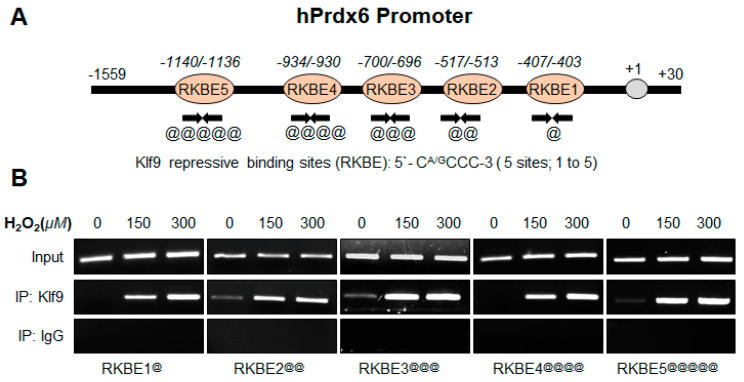
In vivo DNA binding (ChIP) assay disclosed that H_2_O_2_ treatment stimulated Klf9 binding to its new target gene, Prdx6 in dose-dependent fashion. (**A**) Diagrammatic illustration of *Prdx6* promoter containing repressive Klf9 binding element (RKBE). (**B**) hLECs treated with different concentrations of H_2_O_2_ for 3h as indicated. Pulled DNA fragments with Klf9 antibody were subjected to PCR analysis for RKBE site(s) using primers that recognized a fragment of *Prdx6* promoter containing RKBE as indicated. Primers used for amplification of specific region containing Klf9 sites; @ within Klf9 site, @@ within Klf9 site 2, @@@ within Klf9 site 3, @@@@ within Klf9 site 4 and @@@@@ within Klf9 site 5. Control IgG served as negative control.

In this study, we found an inverse correlation between expression levels of Prdx6 and Klf9 (Figure 1, Figure 2, Figure 3 and Figure 4). Furthermore, recently we have reported that aberrant expression of Klf9 in response to lethal doses of SFN represses the antioxidant gene Prdx6 [11]. However, it was not clear whether excessive stress-driven Klf9-dependent ROS amplification was linked to Klf9-mediated transcriptional repression of Prdx6. To achieve this end, hLECs were treated with different concentrations of H_2_O_2,_ as indicated in Figure 8, and performed a ChIP assay to examine Klf9 binding to its RKBE (repressive Klf9 binding element) present in the Prdx6 gene promoter. As shown in Figure 8, Klf9 occupied the Prdx6 gene promoter at RKBEs (5′-C^A/G^CCC-3′), and notably, the occupancy of Klf9 was increased with an increase in oxidative stress driven by the H_2_O_2_ in a concentration-dependent manner. Chromatin immunoprecipitation of H_2_O_2_-treated hLECs with an antibody specific to Klf9 and control IgG antibody showed that Klf9 bound specifically to its repressive site. In the experiment, 10% chromatin was used as input (upper panel), and no amplicon was detected with IgG control (lower panel), demonstrating the specificity of Klf9 antibody binding to Klf9 at RKBE sites (middle panel) present in Prdx6 gene promoter. Collectively, data revealed that Klf9 specifically interacted with RKBE sites present in the *Prdx6* promoter.

### 3.9. Loss and Gain Experiments Disclosed That the Prdx6 Transcription Was Dependent on Cellular Availability of Klf9 during Oxidative Stress

**Figure 9:**
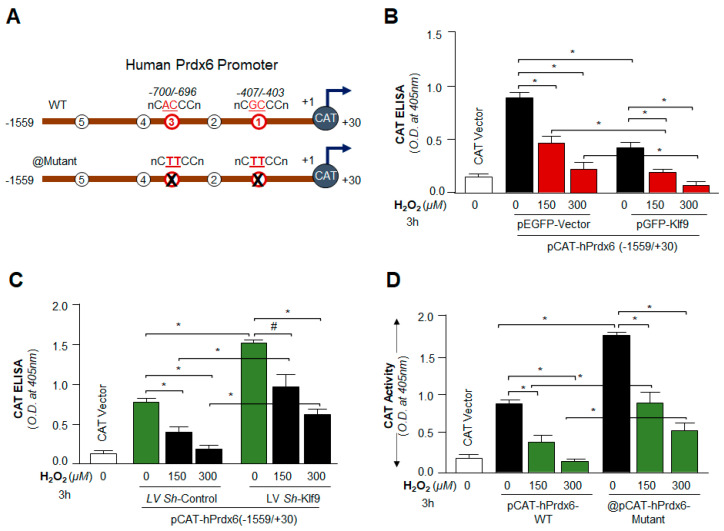
Transactivation assay revealed that Prdx6 transcription was repressed via Klf9 in condition(s) of excessive amount of oxidant. (**A**) Schematic representation of human *Prdx6* promoter [pCAT-hPrdx6 (−1559/+30)] showing Klf9 sites. (**B**) hLECs overexpressing Klf9 displayed a significant reduction in Prdx6 transcription. hLECs were co-transfected with pCAT-hPrdx6 (−1559/+30) WT along with pEGFP-Vector or pGFP-Klf9 plasmid constructs as shown. 48 h later transfectants were exposed to H_2_O_2_ exposure for 3 h as shown, and CAT ELISA was performed to measure the Prdx6 promoter activity. Data are the mean ± S.D. of three independent experiments. * *p* < 0.001; GFP-Klf9 vs. EGFP-Vector transfected cells and H_2_O_2_ treated versus untreated hLECs. (**C**) Transcription assay displayed increased Prdx6 promoter activity in *Klf9*-depleted hLECs. LV *Sh*-Control and/or LV *Sh*-Klf9 infected hLECs were transfected with pCAT-hPrdx6 (−1559/+30) plasmid construct, 48 h later these cells were exposed to H_2_O_2_ as indicated. Prdx6 promoter activity were determined after 3 h of H_2_O_2_ exposure. Data are the mean ± S.D. of three independent experiments. # *p* < 0.05; * *p* < 0.001; LV *Sh*-Klf9 vs. LV *Sh*-Control and H_2_O_2_ treated vs. untreated cells. (**D**) hLECs were transfected with human Prdx6 gene promoter (−1559/+30) linked to CAT wild type (WT) plasmid construct or its mutant at RKBE1 plus RKBE3. 48 h later transfectants were exposed to H_2_O_2_ for 3 h as shown, and CAT ELISA was performed to measure Prdx6 promoter activity. Data are the mean ± S.D. of three independent experiments. * *p* < 0.001 mutant vs. wild-type and H_2_O_2_ treated vs. untreated cells.

As shown in Figure 8, Klf9 occupancy was increased to its sites present in the Prdx6 gene promoter in response to increased oxidative stress. Next, we sought to determine the functionality of H_2_O_2_-induced increased Klf9 binding on Prdx6 transcription by using a transactivation assay, as shown in Figure 9. We engineered *Prdx6* promoter linked to CAT reporter plasmid containing Klf9 sites (Figure 9A), and plasmid mutated at RKBE1 and RKBE3 (RKBE1-mut + RKBE3-mut) sites created by a Site-Directed Mutagenesis (SDM) kit (Figure 9A). hLECs expressing pCAT-Prdx6-WT promoter plasmid along with pEGFP-empty vector or pGFP-Klf9 expression plasmids were exposed to different concentrations of H_2_O_2_ as indicated in Figure 9B. The results revealed that Prdx6 transcription was notably reduced in hLECs overexpressing Klf9 in comparison to the EGFP-empty vector. Perhaps not surprisingly, *Prdx6* promoter activity was further reduced in response to H_2_O_2_-induced stress in a dose-dependent manner in hLECs overexpressing Klf9 (Figure 9B), demonstrating that increased expression of Klf9 was a cause for repression of Prdx6 transcription. Next, we sought to determine whether Klf9 knock down experiment restored the Prdx6 transcriptional activity; we depleted the Klf9 gene by infecting hLECs with *Sh*RNA specific to Klf9 (LV *Sh*-Klf9). hLECs infected with lentiviral *Sh*RNA Control (*LV Sh*-Control) or LV *Sh*-Klf9 were transfected with pCAT-hPrdx6-WT promoter plasmid as shown in Figure 9C and then were exposed to different concentrations of H_2_O_2_. Assay of CAT activity showed increased *Prdx6* promoter activity in *Klf9*-depleted hLECs in comparison to control. Intriguingly, *Klf9*-deficient hLECs facing oxidative stress showed a significant increase in the Prdx6 promoter activity. Collectively, the results demonstrated that Prdx6 transcription is controlled by Klf9 in conditions of oxidative stress. Next, we wanted to validate whether RKBE/Klf9 is specifically involved in the regulation of Prdx6 transcription; we performed Prdx6 transactivation assay by mutating RKBEs in the Prdx6 gene promoter; to achieve this, we mutated repressive Klf9 binding sites (RKBE; C^A/G^CCC) one and three using site-directed mutagenesis (SDM) as shown in Figure 9A and described in Section 2 and our published protocol [11]. Transfectants containing either pCAT-hPrdx6-WT or pCAT-hPrdx6-mutant (mutated at RKBE1 and RKBE3; RKBE1-mutant + RKBE3-mutant) were exposed to different concentrations of H_2_O_2_. We found a significant increase in *Prdx6* promoter activity in the pCAT-hPrdx6-mutant promoter constructs mutated at RKBE site(s) compared to pCAT-hPrdx6-WT containing repressive Klf9 binding sites. Altogether our data demonstrated that RKBE sites present in the Prdx6 gene regulate Prdx6 transcription in the condition of the cellular microenvironment.

### 3.10. A Transcription Inhibitor, Actinomycin D Treatment Demonstrated That Klf9 Induction by Oxidative Stress Did Not Occur Post-Transcriptionally

**Figure 10:**
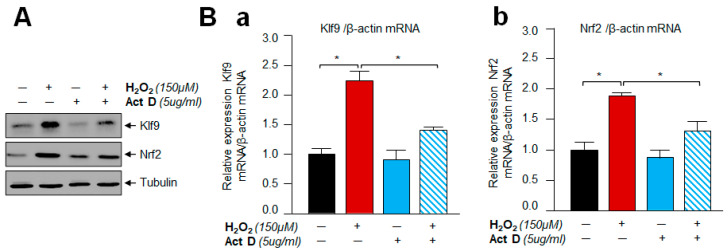
Actinomycin D treatment revealed that Klf9 induction by oxidative stress did not occur post-transcriptionally. (**A**,**B**) hLECs were pretreated with Actinomycin D (Act D) for 30 min followed by H_2_O_2_ for 3 h and proceeded for protein (**A**) and mRNA (**B**) analysis with Klf9 (**Ba**) or Nrf2 (**Bb**) specific probes as indicated. Data are the mean ± S.D. of three independent experiments. * *p* < 0.001 vs. control.

To determine whether Klf9 regulation occurred at the transcriptional or post-transcriptional level, hLECs were incubated with Actinomycin D (5 µg/mL) for 30 min, and then these cells were exposed to H_2_O_2_ as indicated in Figure 10. Expression assays with total protein extract and RNA using an antibody specific to Klf9 demonstrated that Actinomycin D inhibited the H_2_O_2_-induced abundance of Klf9 protein and mRNA expression. The results indicated that activation of Klf9 by H_2_O_2_ does not occur post-transcriptionally, but it was regulated at the transcriptional level.

### 3.11. Under- and Over-Expression Experimentation Disclosed That Klf9 Expression Was Transcriptionally Regulated by Nrf2

**Figure 11:**
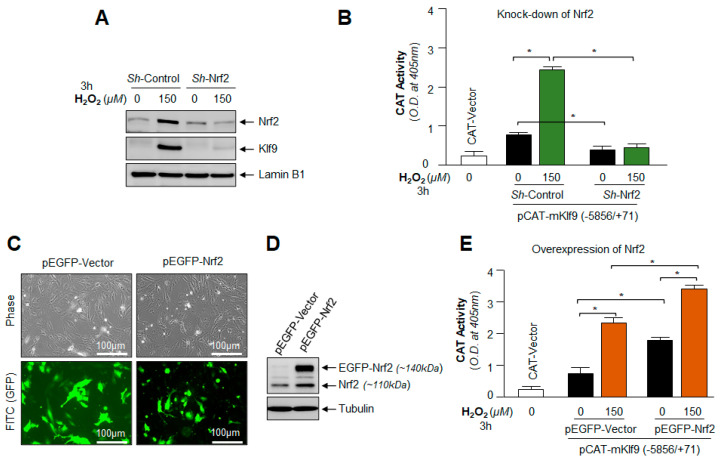
Loss and gain experiments of Nrf2 disclosed that altered Klf9 expression and its promoter activity was directly linked to cellular levels of Nrf2. (**A**,**B**) *Nrf2*-deficient hLECs fails to activate the Klf9 expression and its transcription. (**A**) *Nrf2*-depleted hLECs were exposed to H_2_O_2_ as indicated in Figure 3A for 3 h. Nuclear extract were prepared, and equal amount of protein was immunoblotted using an antibody specific to Nrf2 or Klf9. (**B**) *Nrf2*-deficeint hLECs were transfected with WT *Klf9* promoter (−5856/+71) linked to CAT vector. The transfectants were exposed to H_2_O_2_ for 3 h as shown, and Klf9 promoter activity was measured and shown. Data are the mean ± S.D. of three independent experiments. * *p* < 0.001; H_2_O_2_ treated versus control and *Sh*-Nrf2 vs. *Sh*-Control. (**C**–**E**) Nrf2 overexpression enhanced the Klf9 promoter activity. (**C**) Photomicrograph representing the Nrf2 overexpression in hLECs, left panel: pEGFP-Vector; right panel: pEGFP-Nrf2. (**D**) Western blot analysis showing the Nrf2 overexpression of transfected cells. Cellular extract prepared from pEGFP-Vector and pEGFP-Nrf2 overexpressed hLECs having equal amount of protein was immunoblotted with antibody specific to Nrf2. (**E**) Cells overexpressing Nrf2 showed enhanced Klf9 transcription. hLECs were transfected with *Klf9* promoter (−5856/+71) along with pEGFP-vector and/or pEGFP-Nrf2 plasmids. 48 h later transfectants were exposed to H_2_O_2_ for 3 h, and promoter activity was monitored. Data are the mean ± S.D. of three independent experiments. * *p* < 0.001; H_2_O_2_ treated versus control and pEGFP-Nrf2 vs. pEGFP-Vector.

Next, we asked whether depletion or overexpression of the Nrf2 gene affected Klf9 transcription or expression; hLECs were transiently transfected with *Sh*RNA-Control or *Sh*RNA specific to Nrf2 and generated a stable cell line of hLECs using antibiotic selection pressure. Transfectants were exposed to H_2_O_2,_ and Nrf2 and Klf9 protein expressions in the nucleus were analyzed by immunoblotting, as shown in Figure 11A. We observed that Nrf2 and Nrf2-mediated nuclear accumulation of Klf9 protein in response to H_2_O_2_ treatment in *Sh*-Control hLECs, while Klf9 did not accumulate in the nucleus of Nrf2-depleted hLECs. Data demonstrated that the cellular abundance of Nrf2 was a requisite for Klf9’s aberrant induction and expression. Next, we examined if Nrf2 knock down inhibited Klf9 transcription, *Sh*-Control and *Sh*-Nrf2 hLECs were transfected with pCAT-Klf9 WT promoter construct and then exposed to H_2_O_2_ as shown in Figure 11B, and Klf9 promoter activity was determined. The results demonstrated that Klf9 transcription was significantly augmented in *Sh*-Control hLECs treated with H_2_O_2_, while there was no significant activation of Klf9 transcription in *Sh*-Nrf2 hLECs as shown in Figure 11B.

Furthermore, we next sought to determine the Klf9 status in Nrf2 overexpressed hLECs facing oxidative stress. To this end, hLECs overexpressed with pEGFP-vector or pEGFP-Nrf2 were exposed to H_2_O_2_. Figure 11C displayed transfection efficiency, and Figure 11D showed the levels of Nrf2 in the same transfectants through immunoblotting using an antibody specific to Nrf2. hLECs were transfected with pCAT-Klf9-WT-promoter construct along with pEGFP-Vector or pEGFP-Nrf2 plasmid and exposed to oxidative stress as indicated in Figure 11E. The data revealed that Klf9 transcriptional activity was significantly increased in hLECs overexpressing Nrf2 and that the Klf9 transcriptional activity was further increased in response to oxidative stress. On the whole, data demonstrated that Klf9 is regulated by Nrf2, and Klf9 cellular abundance is directly connected to the increased cellular availability of Nrf2. We surmise that these phenomena that occurred during oxidative stress play a pivotal role in Nrf2-Klf9-dependent regulation of Prdx6 and Prdx6 regulation of ROS levels and biological response(s).

## 4. Discussion

The hormetic response of oxidative stress or electrophilic compounds may be attributed to levels of intracellular ROS, and ROS-driven beneficial or deleterious signaling [78,79,80]. Thus, maintenance of ROS homeostasis is imperative for beneficial biological response in favor of cells health. ROS include H_2_O_2,_ and H_2_O_2_ plays a key role as an intracellular messenger to control cellular physiology in a concentration-dependent fashion [43,81,82,83]. In this study, we intended to uncover the molecular mechanism involved in the regulation and changes of ROS and H_2_O_2_ homeostasis and the molecular events which are responsible for the hormetic effects of ROS. The results from this study, for the first time, showed oxidative stress-driven hormetic effect is regulated via Nrf2-Klf9 regulation of antioxidant response at least in part at the transcriptional level. Our findings suggest that (i) a lower level of oxidative stress (below the threshold levels) results in Nrf2/Prdx6-mediated maintenance of ROS homeostasis and cellular survival while excessive degree (beyond the threshold level) leads to adverse signaling-induced cells injuries; (ii) mechanistically, the changes in intracellular ROS were related to Nrf2-mediated expression and accumulation of Klf9, and Klf9-dependent suppression of Prdx6, resulting in increased ROS levels; (iii) the data also revealed that Nrf2 is transcriptionally regulated Klf9 transcription depending on cellular redox microenvironment; and (iv) Prdx6 expression determined the levels of ROS in response to oxidative conditions via Nrf2-Klf9 activation. It is noteworthy that the intrinsic generation of ROS at a normal physiological level is the major source of oxidants due to the malfunction of antioxidants; however, exposure to extrinsic stresses is an unavoidable factor(s) that can further augment oxidant levels. Recently, adaptive stress responses (hormetic effects), the concept of oxidative stress, and antioxidant responses have been widely accepted. This demonstrates that the lower exposure of prooxidants leads to environmental priming of antioxidant responses, and the process results in better cell survival due to increased antioxidant response [84]. Mechanistically, we reasoned that the presence of mild levels of oxidant during physiological stress within the cellular microenvironment acts as signaling molecules that make cells ready to cope with increased redox challenges, and thus cells survive well under adverse conditions. A similar phenomenon has recently been observed in enhanced survival of neuronal cells; mild/moderate stress applied to cortical neurons decreases the intensity of lipofuscin granules and pathological aggregates as well as in other cell types [85,86]. Moreover, it has been documented that variation in hormetic response is genetically determined, and the hormesis phenomenon plays a pivotal role in maintaining cellular status by regulating cell physiology [87,88,89]. Moreover, many aging-related diseases such as neurodegenerative disease, cancer, cardiovascular disease, cataract, glaucoma, diabetes, etc. are directly or indirectly associated with increased levels of ROS [6,9,90,91,92,93,94,95,96]. To maintain ROS homeostasis, cells are equipped with antioxidant defense systems, containing antioxidant proteins, such as superoxide dismutase (SODs), catalase (CAT), peroxiredoxins (Prdxs), glutathione (GSH), glutathione peroxidase (GPx) [11,12,67,97], however how the antioxidant system goes awry and what molecular events occur, leading to ROS-driven increased cells or tissues damage in response to oxidative stress and with advancing age are still not well explored. In this study, we found that a progressive decline in cellular abundance of Prdx6 expression was directly connected to increased expression of transcriptional proteins Klf9 and Nrf2 with elevated ROS accumulation and subsequent cell death in the aging cells or cells facing oxidative stress (Figure 1). Interestingly, as observed in Figure 1, Figure 2, Figure 3, Figure 4, Figure 5 and Figure 6, the changes in Prdx6 expression were directly connected to the augmented cellular abundance of Nrf2 and Klf9 and the levels of ROS alterations, suggesting that Prdx6 is pivotal to regulate ROS-driven redox signaling. Based on these results, we think that hormetic effects of ROS may be regulated by cellular Prdx6 levels, thereby Prdx6 determines the fate of the cells in cellular microenvironment conditions. Nevertheless, we acknowledge that Prdx6 regulation of ROS levels may not only be linked to modulation in the transcription of Prdx6 but also its posttranslational modifications, specifically, oxidative modification, can play a role in cellular signaling [98,99]. ROS can do so by acting on downstream target proteins such as Prdx6 within the signal transduction pathway to alter their activities via reversible /unreversible oxidation of Cysteine residues [83,100]. However, how Prdx6 posttranslational modification contributes to the regulation of intracellular ROS and thereby in cellular signaling within the cellular microenvironment warrants further investigation.

In previous studies, we have shown that loss of Prdx6 causes an increase in intracellular ROS with increased susceptibility to oxidants-induced cell death, and we also found that other antioxidants were not effective at protecting cells [6,7,9,21,59,65,68,69,71,96,101], suggesting that the role of Prdx6 is pivotal to control ROS homeostasis. Moreover, intracellular ROS generation evoked by oxidative inducers, such as H_2_O_2_ or UV radiation-induced oxidative stress, can damage the cells but also can stimulate the biological process such as cell growth, cell differentiation, and migration of the cells by inducing the Nrf2/ARE-mediated survival pathway [102]. Nonetheless, the hormetic effects of oxidative stress will depend upon levels of intracellular ROS, which are regulated via antioxidant genes such as Prdx6. In this study, we have utilized different concentrations of H_2_O_2_ to uncover the molecular mechanism(s) involved in the controlling hormetic effects of ROS. Our results revealed that relatively increased level of oxidative stress, the level of transcription factor Klf9 was aberrantly expressed and accumulated in the nucleus, and this was directly correlated with increased expression of Nrf2 (Figure 2). Interestingly, we found that paradoxically, Nrf2’s increased expression-mediated Klf9 upregulation, and Klf9-dependent suppression of Prdx6 mRNA and protein and ROS accumulation (Figure 2 and Figure 3). Our results are consistent with studies as reported by other investigators [13,47,51,56].

Furthermore, an investigation of the underlying molecular mechanism(s) involved in Klf9 activation (induction of Klf9) during oxidative stress showed that lower levels of oxidative stress induce the activation of Nrf2 and Nrf2-Prdx6-mediated cellular protection (Figure 2 and Figure 3). In contrast, at higher levels of oxidative stress, Nrf2 is overly accumulated in the nucleus and activates the Klf9 gene transcription, which represses the antioxidant gene expression, such as Prdx6, with a further increase in ROS production (Figure 3). Furthermore, in support of the above observations, our studies also showed that overexpression of Klf9 suppresses Prdx6 expression with increased ROS levels, and these cells were highly susceptible to death in response to oxidative stress. While depletion of the Klf9 gene in hLECs enhanced the Prdx6 gene expression and the transfectants engendered resistance with reduced levels of ROS. These results demonstrate that Nrf2-Klf9 regulates the hormetic effects of ROS via Prdx6 gene transcription (Figure 4 and Figure 5). Moreover, oxidative stress beyond the threshold level induces the Klf9 gene expression. Cells expressing an increased level of Klf9 are found to be over-sensitive to oxidative stress-induced death [11,13]. Klf9-overexpressed NIH3T3 and WI38 cells have been reported to be sensitive to ROS amplification-mediated cell death facing toxic doses of electrophilic compound(s), and the adverse process was related to reduced expression of some antioxidant proteins [13]. Our results of this study revealed that *Klf9*-depleted cells engender resistance against cell death are in agreement with previously reported works [13], wherein the investigators have shown that *Klf9*-depletion provides resistance against oxidants such as paraquat-induced oxidative stress in NIH3T3 and WI38 cells. Furthermore, it has been shown that *Klf9*-deficient mice confer resistance to bleomycin-induced oxidative stress and pulmonary fibrosis. Recently, in studies with other model systems, it has been reported that activated Klf9 increases endoplasmic reticulum (ER)-stress via facilitating calcium release from ER. Conversely, *Klf9*-depletion decreases tunicamycin-induced ER-stress in mouse liver [103]. In addition, *Klf9*-depletion reduces the protease inhibitors bortezomib (BTZ) and carfilzomib (CFZ)-induced ROS levels and leads to ~50% less cell death by enhancing the thioredoxin reductase 2 (Txnrd2) expression in MM1.S and RPMI-8226 cells [104]. These data suggest that the expression level of Klf9 is involved in the regulation of ROS and can determine ROS-dependent biological responses via antioxidant genes regulation, as observed in this study. Recent evidence reveals that in mammalian systems, H_2_O_2_ and other forms of ROS act as secondary messengers in regulating many types of biological functions and physiological activities [105]. Superoxide radical and hydroxyl radical, key members of the ROS class, including less reactive H_2_O_2_, are found to be involved in many cellular processes [106]. Furthermore, the increase in intracellular steady-state levels of ROS due to deficiency of cellular antioxidant(s) can halt prosurvival signaling pathways, suggesting regulation of antioxidant genes and their physiological abundance are required to reverse ROS-mediated cell-death signaling. We recently showed that toxic concentration of SFN can cause aberrant expression and accumulation of Nrf2 in the nucleus, resulting in the repression of antioxidant genes via Klf9 activation [11]. Protease inhibitors BTZ and CFZ induced oxidative stress and enhanced the Klf9 gene expression, which represses the Txnrd2 expression [104]. ROS have hormetic effects on melanin synthesis and melanosome transfer; at high doses, ROS impair melanosome transfer in vitiligo, while low doses promote beneficial proliferation [107]. Additionally, hormetic regulation on angiogenesis as well as on prostaglandin production by ROS has been reported [108,109].

Mechanistically, our studies showed that the regulation of ROS hormesis involves activation of the Nrf2-Klf9-Prdx6 axis; our results of DNA binding coupled with transcriptional assays disclosed oxidative stress-dependent increased activation of Nrf2 (Figure 3C), and its direct binding to ARE present in Klf9 gene promoter and increased transcription (Figure 7, Figure 8 and Figure 9). Similarly, NIH3T3 cells, when exposed to higher concentrations of SFN, showed enhanced Nrf2’s functional binding to ARE sequences present in the *Klf9* promoter leading to Klf9 stimulation-dependent cytotoxicity with suppression of antioxidant gene [13]. This explains the hormetic effects of electrophilic compounds that occur through changes in ROS levels, as found in this study. Furthermore, it has been reported that XBP1 transcriptionally activates Klf9 under high endoplasmic stress (ER) conditions; however, the study did not show whether increased ROS are involved in these phenomena [103]. Nevertheless, our results revealed that Nrf2-induced increased activation of Klf9 was a cause for the repression of Prdx6 transcription. It occurred by Klf9 binding to its repressive binding elements (RKBE) present in some of the antioxidant gene promoters such as Prdx6 [11,13]. On the whole, our findings demonstrate that cellular expression of the Nrf2-Klf9-Prdx6 pathway is regulated at the transcriptional level, wherein Nrf2-Klf9-mediated Prdx6 expression and their cellular abundance play a central role in the regulation of ROS homeostasis, thereby determining ROS-mediated hormetic effects, beneficial or adverse signaling and biological responses. We acknowledge that oxidative stress-induced cellular signaling is complex. This can alter other cellular processes, such as translational as well as posttranslational modification(s) of genes/proteins such as antioxidant genes. These changes in molecules may also affect cellular physiology by changing ROS homeostasis during oxidative stress [24,58,110]. We also recognize that other mechanism (s) or processes could be involved in regulating ROS, such as posttranslational modification of proteins, which needs further investigation; the investigation of such signaling, specifically related to Prdx6 and hormetic effects of ROS are further research line of our laboratory. Nevertheless, our studies suggested that the Nrf2-Klf9-Prdx6 axis can regulate the hormetic effect of ROS at least in part via transcriptional repression of Prdx6 genes in conditions of oxidative stress.

## 5. Conclusions

**Figure 12:**
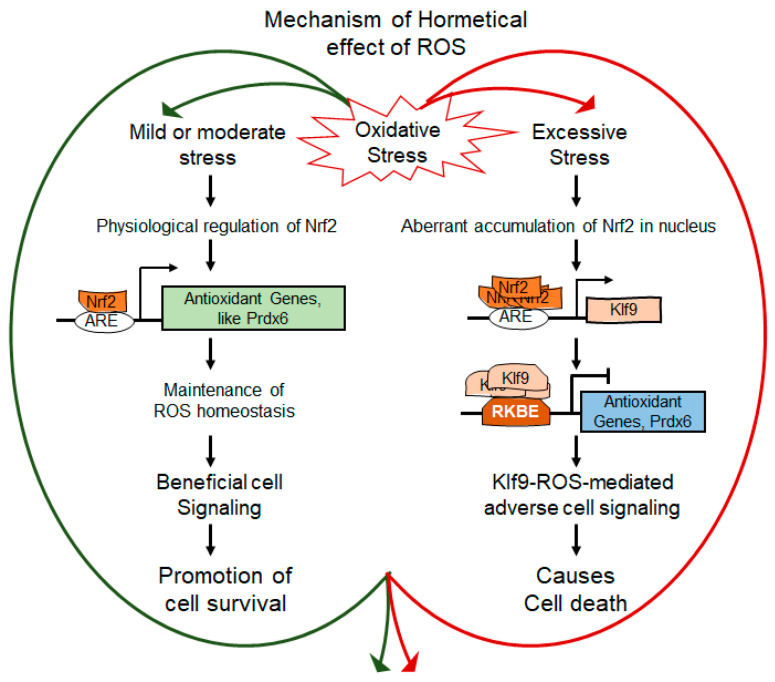
Diagrammatic Illustration of the molecular mechanism governing the bimodal effect of ROS responsible for cellular response in response to oxidative stress. Mild to moderate oxidative stress regulates the Nrf2 at physiological level while excessive oxidative stress aberrantly accumulates the Nrf2 into nucleus. Physiological regulations of Nrf2 activates its target antioxidant genes transcription and expression by binding to antioxidant response element (ARE) sites present in the regulatory region of antioxidant genes and maintain the cellular ROS homeostasis which leads to beneficial cell signaling. At higher levels of oxidative stress, aberrant accumulations of Nrf2 in nucleus, paradoxically activated Klf9 transcription by binding to its ARE sites present in Klf9 gene promoter, and this aberrant Klf9 expression repressed some of the antioxidant genes, such as Prdx6. Finally, these phenomena result in Klf9 abundance-dependent increased ROS amplification, and subsequent cell injuries/death at least in part by transcriptional suppression of Prdx6. Taken together, our data demonstrate that Nrf2-Klf9-Prdx6-axis can play a role in determining ROS-mediated signaling pathway and biological response(s) in conditions of oxidative stress.

In conclusion, our data demonstrated, for the first time, the plausible molecular mechanism of ROS regulation and ROS-mediated hormetic effects during oxidative stress and aging (Figure 12). We found that the hormetic consequence of ROS was regulated at least in part by transcription suppression of Prdx6 and was connected to Nrf2-Klf9’s cellular status. However, predicting Nrf2’s beneficial or harmful effect is challenging since it is dose-dependent on inductors. Thus, it is highly imperative to take into consideration and to target the factor(s) responsible for harmful signaling during Nrf2 activation. Because our results revealed that Klf9 upregulation due to aberrant Nrf2 activation causes ROS amplification and subsequent cell death (Nrf2-Prdx6-mediated survival pathway turns to death pathway) in response to excessive oxidative stress, and the fact that these events are at the transcriptional level, we propose that the application of Klf9 *Sh*RNA and/or Klf9 inhibitor(s) would be a possible strategy to blunt or treat age-associated disorders related to increased ROS-induced injurious signaling. However, future studies are warranted to develop Klf9 antagonists and determine the consequences that the hormetic effects of ROS may have on other physiological functions.

## Data Availability

No supporting Data.
